# Transcriptional Profiling of *Mycobacterium tuberculosis* Replicating *Ex vivo* in Blood from HIV- and HIV+ Subjects

**DOI:** 10.1371/journal.pone.0094939

**Published:** 2014-04-22

**Authors:** Michelle B. Ryndak, Krishna K. Singh, Zhengyu Peng, Susan Zolla-Pazner, Hualin Li, Lu Meng, Suman Laal

**Affiliations:** 1 Department of Pathology, New York University Langone Medical Center, New York, New York, United States of America; 2 Institutes of Biomedical Sciences, Shanghai Medical College, Fudan University, Shanghai, China; 3 Veterans Affairs New York Harbor Healthcare System, New York, New York, United States of America; 4 Center for Virology and Vaccine Research, Beth Israel Deaconess Medical Center, Harvard Medical School, Boston, Massachusetts, United States of America; University of Delhi, India

## Abstract

Hematogenous dissemination of *Mycobacterium tuberculosis* (*M. tb*) occurs during both primary and reactivated tuberculosis (TB). Although hematogenous dissemination occurs in non-HIV TB patients, in ∼80% of these patients, TB manifests exclusively as pulmonary disease. In contrast, extrapulmonary, disseminated, and/or miliary TB is seen in 60–70% of HIV-infected TB patients, suggesting that hematogenous dissemination is likely more common in HIV+ patients. To understand *M. tb* adaptation to the blood environment during bacteremia, we have studied the transcriptome of *M. tb* replicating in human whole blood. To investigate if *M. tb* discriminates between the hematogenous environments of immunocompetent and immunodeficient individuals, we compared the *M. tb* transcriptional profiles during replication in blood from HIV- and HIV+ donors. Our results demonstrate that *M. tb* survives and replicates in blood from both HIV- and HIV+ donors and enhances its virulence/pathogenic potential in the hematogenous environment. The *M. tb* blood-specific transcriptome reflects suppression of dormancy, induction of cell-wall remodeling, alteration in mode of iron acquisition, potential evasion of immune surveillance, and enhanced expression of important virulence factors that drive active *M. tb* infection and dissemination. These changes are accentuated during bacterial replication in blood from HIV+ patients. Furthermore, the expression of ESAT-6, which participates in dissemination of *M. tb* from the lungs, is upregulated in *M. tb* growing in blood, especially during growth in blood from HIV+ patients. Preliminary experiments also demonstrate that ESAT-6 promotes HIV replication in U1 cells. These studies provide evidence, for the first time, that during bacteremia, *M. tb* can adapt to the blood environment by modifying its transcriptome in a manner indicative of an enhanced-virulence phenotype that favors active infection. Additionally, transcriptional modifications in HIV+ blood may further accentuate *M. tb* virulence and drive both *M. tb* and HIV infection.

## Introduction

Infection with *M. tuberculosis* (*M. tb*) is initiated by the few bacilli in a droplet that are inhaled into the alveolus, which results either in establishment of infection and progression to primary TB or, by 4–5 weeks post-infection, elicitation of immune responses that control bacterial replication and result in latent TB. Studies in animal models have demonstrated hematogenous dissemination of *M. tb* from the lungs to various organs, including lymph nodes, spleen, liver, pancreas, adrenal, and heart, as well as reseeding of the lung, during primary infection [1], [2], [3]. Studies performed in the pre-ATT (anti TB treatment) era demonstrated hematogenous dissemination of *M. tb* in a majority of pediatric cases of primary TB [4]. Viable *M. tb* have been demonstrated in lungs, liver, spleen and kidneys of healthy individuals who died of causes unrelated to TB, in a TB endemic setting [5]. This again provides evidence of hematogenous dissemination of *M. tb* during primary infection resulting in latent TB [5]. Bacteremia in TB patients with pulmonary TB has been demonstrated for several decades [4], [6], [7]. Evidence for disseminated *M. tb* in patients who reactivated their latent infection also exists [8]. Thus, bacteremia and hematogenous dissemination of *M. tb* is an important component of the natural course of establishment of infection and progression to both primary and reactivated TB.

TB is one of the most common infections in HIV-infected (HIV+) individuals in TB endemic countries, where 60–70% of the HIV+ patients develop TB [9]. Co-infected individuals are at a ∼20–40 fold higher risk for TB [10], and manifestation of TB is independent of CD4+ T cell status and peripheral viral load [11], [12], [13], [14], [15]. Extrapulmonary TB (EPTB), disseminated TB and miliary TB occurs in over 50% of the HIV+ TB patients [16], [17], [18], [19], [20]. *M. tb* bacteremia is common in disseminated TB, and has been increasingly reported in TB endemic regions [21], [22], [23], [24], [25], [26].

Despite the importance of hematogenous dissemination during for establishment of latent TB, or progression to active disease, how *M. tb* adapts to the blood environment to remains to be investigated. Other pathogens such as *Neisseria meningitidis, Listeria monocytogenes*, and Group A and Group B Streptococcus modulate their transcriptional profiles during bacteremia [27], [28], [29], [30]. Considering the exquisite sensitivity of *M. tb* to its environment and its ability to adapt to different environments, it is likely that *M. tb* also modulates its transcriptome during bacteremia. Transcriptome changes have been described during adaptation of *M. tb* to environments that may mimic *in vivo* conditions within the granuloma or the phagosome [31], [32], [33], [34], [35], [36]. *M. tb* also modifies its transcriptional profile during adaptation to different intracellular environments, [37], [38]. Interestingly, differences in *M. tb* transcriptomes during infection of immunocompetent (BALB/c) and immunocompromised (SCID) mice has also been reported [39]. Differential gene expression has been reported in bacteria from different portions of the same granuloma, and in non-granulomatous regions of the lung [40]. Thus, *M. tb* may also discriminate between HIV- healthy and HIV+ immunodeficient individuals during bacteremia.

We have investigated the transcriptome of *M. tb* replicating in human whole blood to investigate the adaptation during bacteremia. To determine how *M. tb* discriminates between the blood environment of immunocompetent and immunodeficient individuals, we have compared the transcriptional profile during growth and replication in blood from HIV- and HIV+ donors. Our results demonstrate that *M. tb* not only survives and replicates in blood from both HIV- and HIV+ donors, it also enhances its virulence/pathogenic potential in blood, more emphatically in blood from HIV+ patients. The blood-specific transcriptome reflects down-regulation of genes required for survival in dormancy, induction of cell-wall remodeling, alteration in mode of iron acquisition, potential evasion of immune surveillance, and enhancement in the expression of important virulence factors that drive active *M. tb* infection and dissemination. Importantly, these changes are accentuated during replication in blood from HIV+ patients. Our results also demonstrate that *esat6*, the gene encoding ESAT-6, which plays an important role in dissemination of *M. tb* from the lungs, is upregulated in *M. tb* growing in blood [41], [42], especially in bacteria growing in HIV+ patient blood. Preliminary experiments suggest that purified recombinant ESAT-6 promotes HIV replication in U1 cells and could serve to directly accelerate the progression of HIV-infection.

## Results and Discussion

### Transcriptome analysis of *M. tb* in whole blood from HIV- and HIV+ humans

**Figure 1:**
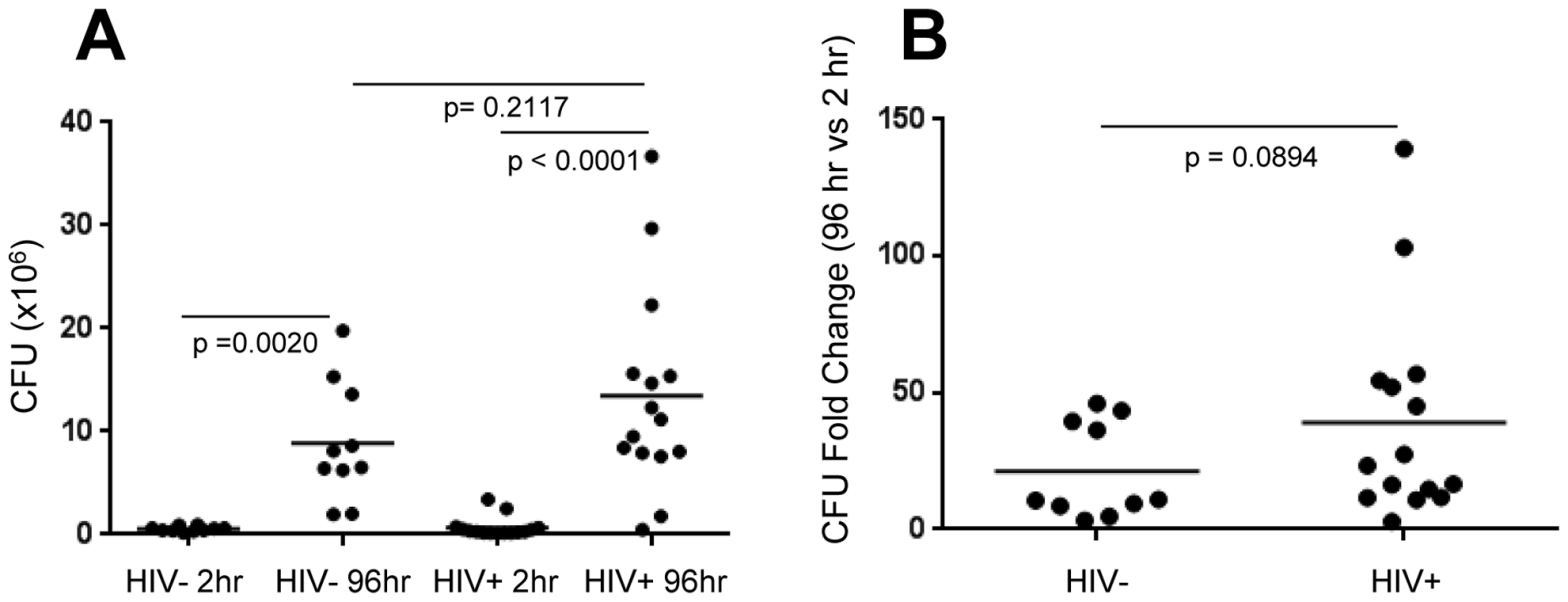
*M. tb* replication in whole blood from HIV- and HIV+ subjects. (A) Total (intra- and extra-cellular) *M. tb* CFU counts after 2 and 96 hr in blood from 10 HIV- donors or in blood from 15 HIV+ patients. (Wilcoxon Mann-Whitney two-tailed analysis; P values≤0.05 statistically significant) (B) *M. tb* CFU fold change between 2 hr and 96 hr in blood from HIV- donors and HIV+ patients (same as in panel A). (Mann-Whitney two-tailed analysis; P value<0.05 statistically significant).

To investigate the transcriptome of *M. tb* in the hematogenous environment, we first evaluated the ability of *M. tb* H37Rv to survive and replicate in fresh whole blood from 10 HIV- and 15 HIV+ donors (Figure 1; TABLE S1). Significant bacterial growth was observed within 96 hr post-inoculation in blood from both HIV- and HIV+ donors (p values = 0.0020 and <0.0001, respectively). There was no difference in bacterial replication in the two blood environments (p value = 0.2117) Fig 1A). At 96 hours post- inoculation, there was 3–46 fold (mean 21 fold) increase in CFU in the blood from the 10 HIV- donors (Figure 1B). The trend of *M. tb* replication was higher in blood from HIV+ donors (3–139 fold increase; mean = 39 fold) but the difference was not statistically significant (Figure 1B). This is in contrast to earlier studies where replication of BCG in whole blood from HIV- and HIV+ children was studied, and the bacterial replication was significantly higher in the latter environment. Whether the difference in these results is due to use of BCG compared to H37Rv, or differences in the pediatric and adult hematological environment is not known [43]. While *M. tb* replication in 7H9 broth over 96 hr has been demonstrated in a number of previously published studies, e.g., [44], [45], the dilution of blood samples with glutamine- and heparin- supplemented RPMI is unlikely to promote *M. tb* replication since *M. tb* H37Rv failed to grow in the medium alone (data not shown). Moreover, the same dilution media was used in both HIV- and HIV+ blood cultures.

**Figure 2:**
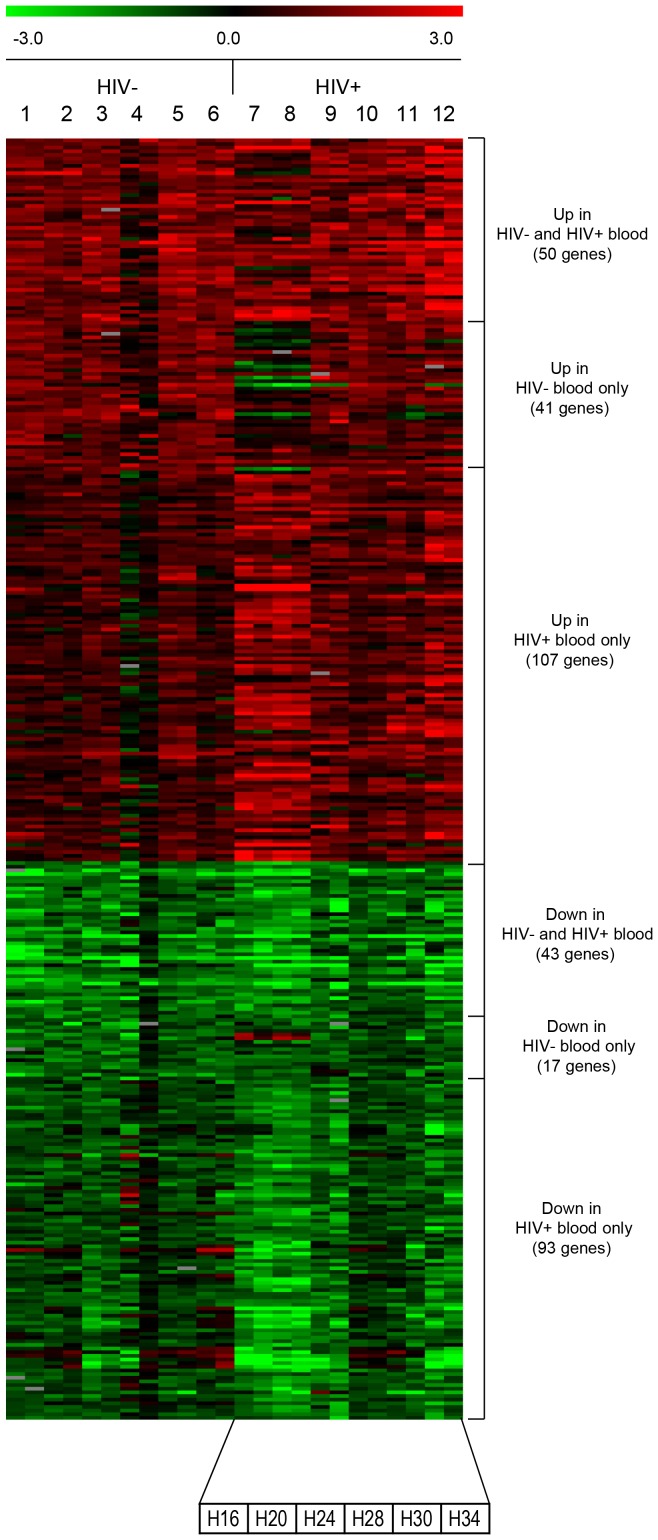
Differentially expressed *M. tb* genes in whole blood from HIV- and HIV+ donors. Heat map denoting upregulated genes in red and down-regulated genes in green in *M. tb* grown in blood from 6 HIV- donors (1-6) and 6 HIV+ patients (7-12) with dye flip (12 samples for each condition, HIV- and HIV+). Genes are listed as upregulated or down-regulated in blood from both HIV- and HIV+ donors or in HIV- donor blood only or HIV+ patient blood only. HIV+ blood sample codes (H#) are listed at the bottom for comparison with CD4+ T cell counts and viral loads in Table S1.

Following confirmation that human blood from both HIV- and HIV+ donors supports bacterial survival and replication *ex vivo* (Figure 1A), the transcriptional profile of *M. tb* replicating in blood from 6 HIV- individuals and 6 HIV+ individuals was investigated with bacteria grown in 7H9 bacterial media (Figure 2). (CD4+ T cell numbers and viral load for the 6 HIV+ individuals is provided in TABLE S1).

Analysis of the transcriptional profile of *M. tb* during replication in blood from HIV- donors revealed that 91 genes were upregulated and 60 were down-regulated (Figure 2). In contrast, in bacteria replicating in blood from HIV+ patients, 157 genes were upregulated and 136 down-regulated (Figure 2). Thus, a more extensive transcriptional adaptation was observed in *M. tb* replicating in the HIV+ blood environment. Of the differentially expressed genes, 41 were upregulated and 17 down-regulated only in blood from HIV- donors (Figures 2 and S1) and 107 upregulated and 93 down-regulated only in blood from HIV+ patients (Figures 2 and S2). Fifty upregulated and 43 down-regulated genes overlapped in the two blood environments, indicating a “blood-specific” transcriptional profile (Figures 2 and S3). Although the presence of heparin in the blood could contribute to the differential expression of some *M. tb* genes, these would be common for both the blood environments. Thus, *M. tb* can adapt to the blood environment and can also distinguish the blood environments of HIV- and HIV+ donors (see TABLE S2 and TABLE S3 for lists of all upregulated and down-regulated *M. tb* genes in whole blood from HIV- and HIV+ donors, respectively). Unsupervised hierarchical clustergrams for all upregulated (Figure S4A) and down-regulated (Figure S4B) genes produced 10 clusters for the former and 6 clusters for the latter genes. As expected, *M. tb* genes differentially expressed only in blood from HIV- donors or from HIV+ patients predominantly clustered separately from each other, while genes differentially expressed in both environments were spread across clusters.

**Figure 3:**
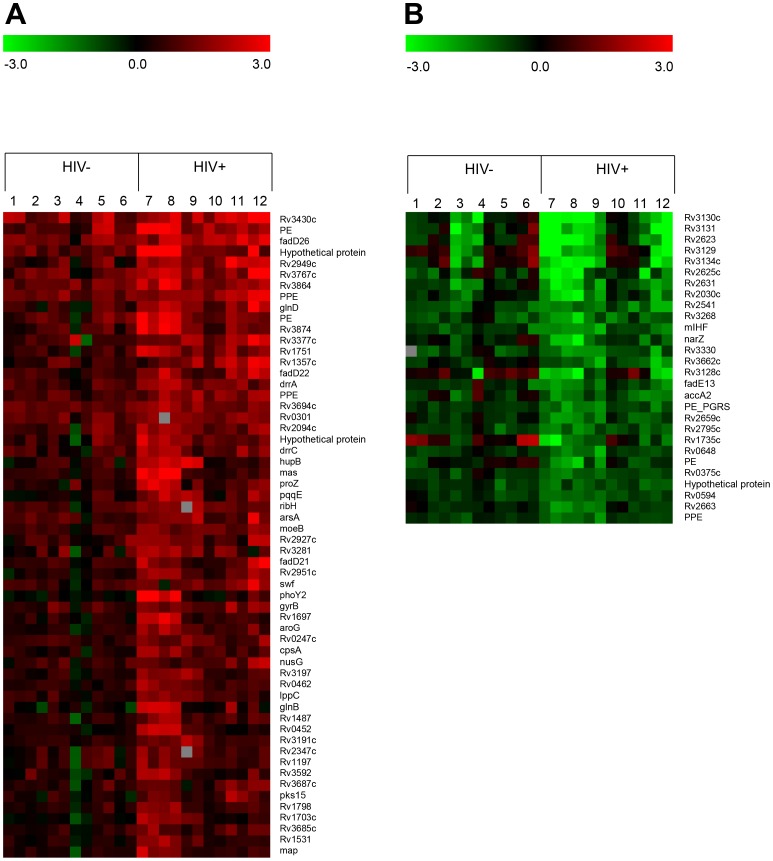
Differential *M. tb* gene expression in blood from HIV+ patients versus HIV- donors. (A) Heat map of *M. tb* genes upregulated ≥2 fold during replication in blood from HIV+ patients (7–12) versus HIV- donors (1-6). (B) Heat map of *M. tb* genes down-regulated ≥2 fold during replication in blood from HIV+ patients (7-12) versus HIV- donors (1-6).

For further verification of the differences in transcriptional profiles during replication in blood from HIV+ versus HIV- donors, the log2 ratio profiles obtained from the blood/7H9 microarray analyses were statistically evaluated (see Materials and Methods) and *M. tb* genes exhibiting a ≥2 fold difference between the two environments identified (Figure 3). Based on this analysis, 58 genes were upregulated and 28 down-regulated during growth in HIV+ patient blood compared to HIV- donor blood (Figure 3). The similarities between the transcriptional profiles in HIV- and HIV+ blood, as well as the accentuated regulation in blood from HIV+ donors is evident in Figure 3.

### Functional distribution of *M. tb* genes differentially expressed in blood from HIV- and HIV+ donors

**Figure 4:**
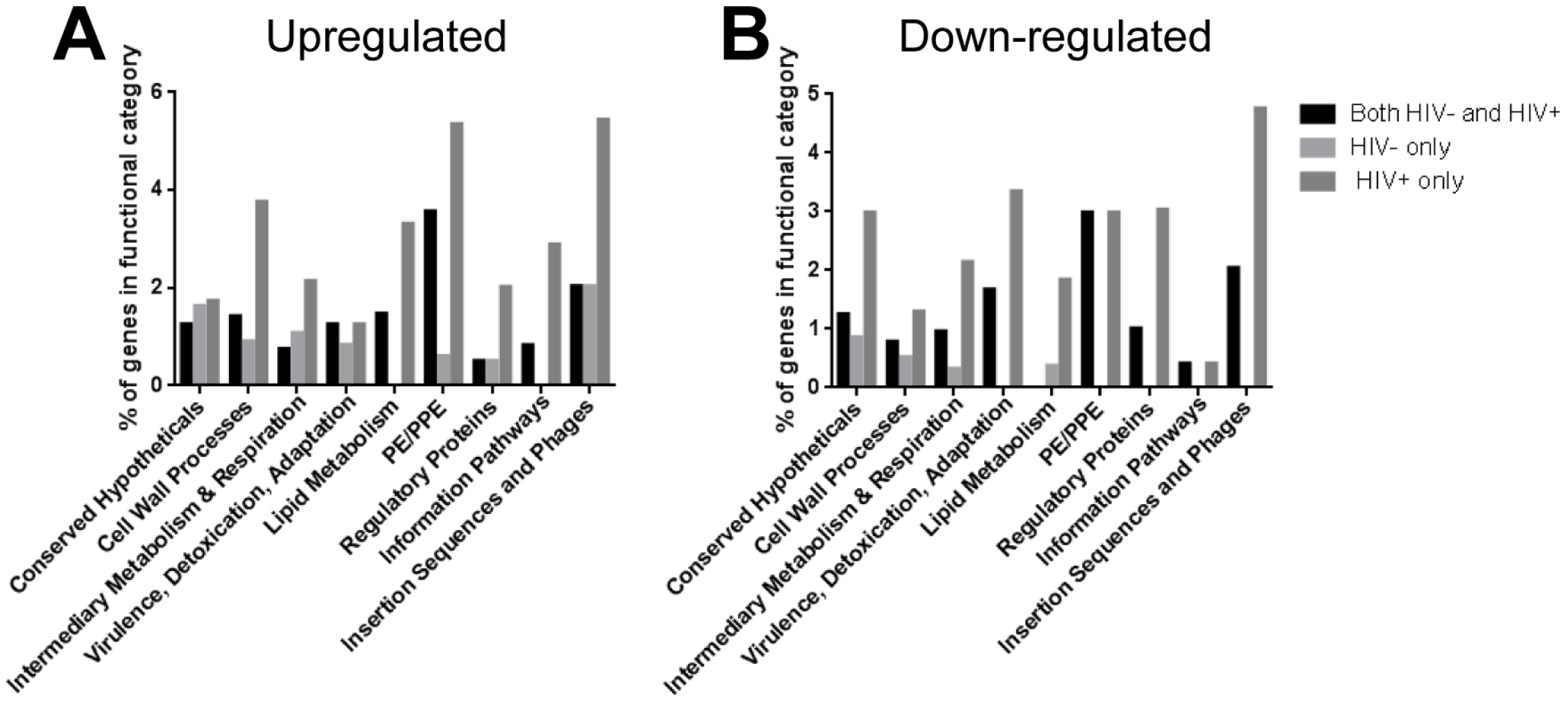
Percentages of genes within functional categories differentially expressed in blood from HIV- and/or HIV+ donors. (A) Upregulated genes arranged by functional category and corresponding blood environments. (B) Down-regulated genes arranged by functional category and corresponding blood environments. (Functional categories as designated in the Tuberculist website http://tuberculist.epfl.ch/)

The *M. tb* genes differentially expressed in the blood environment were categorized based on up- and down-regulation, in HIV- or HIV+ blood environments or both, and by functional category (http://tuberculist.epfl.ch/index.html; TABLE 1, Figure 4A,B). All functional categories exhibited some degree of differential regulation during replication in the blood (TABLE 1); however, Hypergeometric Distribution and Fisher's Exact testing did not reveal any particular functional category as being significantly enriched in blood from either HIV- or HIV+ donors (data not shown). Assessment of the proportion of differentially expressed genes in any particular functional category showed that genes annotated in the “insertional sequences and phages” (16.3%), “PE/PPE” (15.5%) and “conserved hypotheticals” (9.7%) were the top three functional classes affected; the roles of these classes of genes in *M. tb* are largely unknown. The next most regulated functional categories were “cell-wall and cell-wall processes” (8.7%), and “virulence, detoxification, adaptation” (8.4%). Slightly less regulated were “intermediary metabolism and respiration” (7.4%), “regulatory proteins” (7.1%), and “lipid metabolism” (7.0%), and the least regulated was the “information pathways” (4.5%) category. Based on the percentage of genes affected within a functional category, the most upregulated categories (Figure 4A), aside from “insertional sequences and phages”, were “PE/PPE”, “lipid metabolism” and “cell-wall processes”. Similarly, the most down-regulated categories (Figure 4B) include “insertional sequences and phages” and “PE/PPE”, as well as “virulence, detoxification, adaptation” and “conserved hypotheticals”. Interestingly, in nearly all functional categories, the numbers of genes differentially expressed (both upregulated and down-regulated) were higher in *M. tb* replicating in blood from HIV+ donors (Figure 4) emphasizing the more extensive transcriptional adaptation of *M. tb* to the HIV+ blood environment.

**Table 1:** Numbers of *M. tb* genes differentially expressed in blood from HIV- and/or HIV+ subjects arranged by functional category.

Functional Category^a^	Number of Genes Differentially Regulated in Blood						
	HIV- only		HIV+ only		Both HIV- and HIV+		
	Up	Down	Up	Down	Up	Down	Total
Conserved Hypotheticals (1042)^b^	17	9	18	31	13	13	101 (9.7%)^c^
Cell Wall and Cell Processes (772)	7	4	29	10	11	6	67 (8.7)
Intermediary Metabolism & Respiration (936)	10	3	20	20	7	9	69 (7.4%)
Virulence, Detoxification, Adaptation (239)	2	0	3	8	3	4	20 (8.4%)
Lipid Metabolism (272)	0	1	9	5	4	0	19 (7.0%)
PE/PPE (168)	1	0	9	5	6	5	26 (15.5%)
Regulatory Proteins (198)	1	0	4	6	1	2	14 (7.1%)
Information Pathways (242)	0	0	7	1	2	1	11 (4.5%)
Insertion Sequences and Phages (147)	3	0	8	7	3	3	24 (16.3%)
Total	41	17	107	93	50	43	351

a Functional categories as designated in the Tuberculist website (http://tuberculist.epfl.ch/index.html).

b Numbers in parentheses indicate total number of genes in the category in the *M. tb* H37Rv genome.

c Percentages in parentheses next to “Total” numbers indicate the percentage of genes differentially expressed within the indicated category.

### *M. tb* dormancy-related transcriptome is suppressed during replication in whole blood

**Figure 5:**
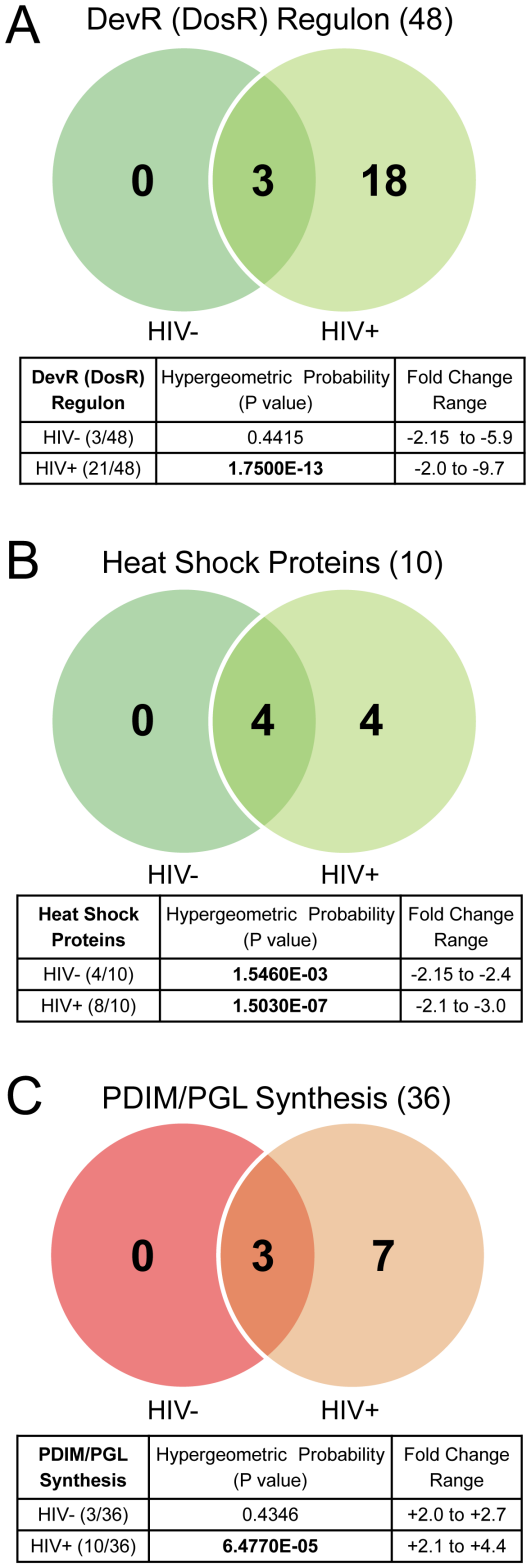
Specific categories of *M. tb* genes affected during growth in the blood environment. (A) DosR regulon genes (B) Genes encoding heat shock proteins (8) and stimulators of heat shock proteins (2) (Total 10 genes assessed). (C) Genes involved in PDIM and PGL synthesis and transport. Venn diagrams indicate numbers of genes differentially expressed in blood from HIV- and/or HIV+ donors with common affected genes in the overlap. (Green indicates down-regulated. Red indicates upregulated.) Numbers in parentheses next to headings indicate the total number of genes in the designated category encoded in the *M. tb* genome. Tables below include the Hypergeometric Probability P-values from Fisher's Exact testing of differentially expressed genes represented in each category and each blood environment (HIV- or HIV+) (significant P-values in bold), as well as the ranges of expression fold change.

*M. tb* dormancy is characterized by a viable, low metabolic, anaerobic, non-replicating state associated with latent tuberculosis infection [46]. The *M. tb* DevR (DosR) regulon consists of ∼50 genes induced during adaptation to multiple stressful environments, including hypoxia encountered within granulomas [35], [47] and reactive nitrogen intermediates derived from activated macrophages [48]. Such stresses can lead to inhibition of aerobic respiration, suppression of bacterial replication, and *M. tb* transition into a dormant state, contributing to latency [46]. The DevR (DosR) response contributes to survival of *M. tb* under these stresses and in the dormant state [49]. Compared to broth-grown bacteria, several DevR (DosR) regulon genes were down-regulated in *M. tb* replicating in whole blood (Figure 5A). Thus, Rv1812c, Rv1813c, and Rv2031c (*hspX*), all predicted to be in the DevR (DosR) regulon, were down-regulated in blood obtained from both HIV- and HIV+ donors; an 18 DevR (DosR) regulon genes were down-regulated in bacteria growing only in the blood from HIV+ patients. Fisher's Exact testing revealed significant enrichment of this regulon among down-regulated genes during replication in blood from HIV+ patients but not in blood from HIV- donors (Figure 5A), and 11 of the 28 genes down-regulated by *M. tb* in blood from HIV+ patients versus HIV- donors (Figure 3) belong to the DevR (DosR) regulon. Since the same logarithmically broth-grown *M. tb* reference RNA was used across all microarray experiments, the extensive down-regulation of this regulon in HIV+ blood is not an artifact of the reference RNA. The vast majority of the differentially expressed DevR (DosR) regulon genes (19/21) group in hierarchical Cluster #6 (Figure S4B), and of the 61 genes in this cluster, 53 are down-regulated in blood from HIV+ patients only (Figure S4B).

In addition to the DevR (DosR) regulatory system, the MprAB (mycobacterial persistence regulator two-component system) also participates in the establishment and maintenance of “persistence” during dormant *M. tb* infection [50]. The virulence-associated operon encoding Rv1812c and Rv1813c, regulated by DevR (DosR) and down-regulated in the blood environment, is also regulated by the MprAB regulatory system [51]. Rv0982, the gene encoding the sensor kinase of the system, MprB, is down-regulated in *M. tb* replicating in blood from both HIV- and HIV+ donors; in addition, Rv0981, encoding the response-regulator, MprA, is down-regulated only in blood from HIV+ patients. Finally, Rv1009, the gene encoding probable resuscitation-promoting factor RpfB, believed to promote the resuscitation and growth of dormant, non-growing bacteria [52], is upregulated during growth in blood from HIV+ patients. Together these results suggest that the blood environment does not produce stresses that lead to inhibition of *M. tb* growth and transition to dormancy, implying permissiveness for active bacterial replication. Moreover, in addition to preventing entrance into dormancy, the HIV+ patient blood environment may also promote exit from the dormant state.

### Stress responses of *M. tb* in the blood environment

Transcripts for genes involved in global stress responses, e.g. genes encoding RelA, universal stress proteins, sigma factors etc., were either unaffected or down-regulated during replication in whole blood. However, transcripts for Rv2429 (*ahpD*), involved in the *M. tb* oxidative stress response, were upregulated in blood from both HIV- and HIV+ donors; Rv2428 (*ahpC*) and Rv0462 (*lpd*) were additionally upregulated in the latter environment. AhpD has mild hydroperoxidase activity, and also functions, in conjunction with Lpd and Rv2215 (DlaT) to reduce AhpC, an alkyl hydroperoxidase which detoxifies organic peroxides [53], hydrogen peroxide (H_2_O_2_) [54], and peroxynitrate [55], indicating the bacterial requirement for this antioxidative/antinitrosative system during replication in blood. AhpC expression is repressed in *M. tb* during *in vitro* broth culture, in macrophages and in mouse lungs and spleen by an unknown regulator [56]. The derepression of the AhpC system, and the upregulation of *aphD, lpd*, and *aphC* in the HIV+ patient blood without any changes in expression of the *katG* gene suggests that *M. tb* encounters source(s) of oxidative/nitrosative stress that may not be neutralized by KatG.

Oxidative stress can be generated endogenously as free radical byproducts of aerobic respiration accumulate [57], while exogenous sources of oxidative stress can be experienced through interactions with activated macrophages and neutrophils [57]. Thus, transcripts for Rv0249c, a succinic dehydrogenase involved in the interconversion of fumarate and succinate, and Rv2200c (*ctaC*), a probable transmembrane cytochrome C oxidase, both involved in aerobic respiration were upregulated in blood from both HIV- and HIV+ donors while genes involved in an alternative respiratory pathway when aerobic respiration is inhibited e.g. Rv0392c (*ndhA*), Rv1854c (*ndh*), Rv1162 (*narH*) and Rv1164 (*narI*) were not affected. Another potential source of oxidative stress for *M. tb* is an environment which is high in free iron since iron drives the Fenton reaction which produces DNA damaging active radicals [58]. As discussed below, based on the transcriptional profile of *M. tb* in the blood environment, excess free iron may be another plausible contributor to oxidative stress.

Another source of oxidative stress as well as nitrosative stress is the intra-phagosomal environment of activated macrophages where reactive species such as H_2_O_2_, superoxide (O^2^-), and nitric oxide (NO) are encountered [59]. Of these, NO represses *M. tb* replication and the aerobic pathway [48], [60], and low concentrations of NO are known to initiate a transcriptional response in *M. tb* characterized by the upregulation of the DevR (DosR) regulon [48]. As discussed above, several of the DevR (DosR) regulon genes were down-regulated during replication in blood (Fig 5A). Also, the DevR (DosR)-regulated Rv1737c and Rv1736c (*narK2X*) operon was down-regulated in the HIV+ patient blood environment. Rv1737c (NarK2) is involved in nitrate reduction when *M. tb* is in a senescent stationary phase, as well as the transport of nitrate in and nitrite out of the bacterium. Only low levels of *narX* and *narK2* transcripts are detectable in aerobic *M. tb* cultures [61]. Furthermore, nitrate reduction is associated with the respiratory switch under hypoxic conditions [62] and has also been shown to protect against acidic and nitrogen species stresses, conditions associated with the intra-macrophage environment [63]. The down-regulation of *narX* and *narK2* in HIV+ patient blood is consistent with the favoring of *M. tb* replication and aerobic respiration in this environment. Stresses which lead to misfolding of bacterial proteins activate a “heat shock response” in *M. tb* [34], [47], [64]. *M. tb* encodes eight heat shock proteins (HSPs) which function as molecular chaperones [65]. Transcripts encoding HSPs Rv0384c (*clpB*), Rv0251c (*hsp*), Rv2031c (*hspX*), and Rv0440 (*groEL2*) were down-regulated in blood from both HIV- and HIV+ donors, and transcripts for HSPs, Rv3418c (*groES*) and Rv0350 (*dnaK*) were additionally down-regulated in HIV+ patient blood (Figure 5B). Moreover, Rv0352 (*dnaJ*) and Rv0351 (*grpE*), both encoding proteins that are stimulators of DnaK activity, were also down-regulated in HIV+ patient blood (Fig 5B). The down-regulation of genes encoding heat shock proteins as a group is statistically significant by Fisher's Exact test in both blood environments (Figure 5B), and suggests lack of stresses affecting protein folding during growth in blood. HSPs are also highly immunogenic [66] and proinflammatory [67], and overexpression of *M. tb* HSPs reduces bacterial survival in mice lungs due to enhanced immune recognition [68]. Consequently, HSPs are considered potentially useful in the development of vaccines and immunotherapy against TB [69], [70]. The dampening of the “heat shock response” could indicate evasion of *M. tb* from the host immune surveillance.

### *M. tb* cell-wall remodeling

The *M. tb* cell-wall is a complex structure serving as the interface of the host and the pathogen, and is important for *M. tb* survival and virulence [71]. The transcriptome of *M. tb* growing in whole blood suggests that significant *M. tb* cell-wall remodeling occurs in this environment. Specifically, the phthiocerol dimycocerosate (PDIM) and phenol glycolipid (PGL) synthesis pathway genes were particularly represented amongst the upregulated cell-wall synthesis genes and statistically enriched in blood from HIV+ patients (Figure 5C). PDIM and PGL are closely related surface-exposed cell-wall components present only in pathogenic mycobacteria and have been associated with virulence in animal models [72]. Thus, transcripts for Rv2930 (*fadD26*), Rv2939 (*papA5*) and Rv2953, all involved in PDIM biosynthesis, were upregulated in blood from both HIV- and HIV+ donors. In addition, transcripts for Rv2936 (*drrA*) and Rv2938 (*drrC*), both of which are involved in the active transport of PDIM across the *M. tb* membrane were also upregulated in the HIV+ patient blood, as was Rv2940c (*mas*) which is involved in the elongation of mycocerosyl lipids, components of both PDIM and PGL. Together these results provide strong evidence that PDIM synthesis and transport by *M. tb* is promoted in blood from HIV+ donors. This suggests that *M. tb* can enhance its virulence during replication in blood from HIV+ patients.

Transcripts for Rv2947c (*pks15/1*), Rv2948c (*fadD22*), and Rv2950c (*fadD29*), all of which are involved specifically in the synthesis of PGL, were upregulated in *M. tb* H37Rv exclusively in HIV+ patient blood. PGL has been associated with “hyperlethality” of *M. tb* HN878 [73] and correlates with increased bacterial burden and dissemination in the rabbit CNS infection model [74]. While the *M. tb* H37Rv cell-wall lacks PGL due to a deletion mutation in the *pks15* gene which disrupts the PGL-specific synthesis pathway [75], the potential upregulation of these PGL synthesis genes in clinical *M. tb* strains which harbor a fully functional Pks15/1 could result in a more aggressive phenotype.

Besides the genes involved in PDIM/PGL synthesis, 6 *pe/ppe* genes were upregulated and 5 down-regulated in bacteria replicating in blood from both HIV- and HIV+ donors; an additional 9 *pe/ppe* genes were upregulated and 5 down-regulated in HIV+ patient blood only (TABLE 1 and Figure 4). Only one *pe/ppe* gene was exclusively upregulated in HIV- donor blood. PE/PPE proteins are found only in mycobacteria, more commonly in pathogenic species, and several are demonstrated to be cell-wall proteins that contribute to virulence and pathogenesis [76], [77], [78]. The differential regulation of this class of proteins in the human blood, and more extensively in HIV+ patient blood, is further indication of cell-wall remodeling in this environment that may alter the virulence of *M. tb.*

### Upregulation of ESX-1 and ESX-5 type VII secretion systems and genes encoding ESAT-6-like proteins

**Figure 6:**
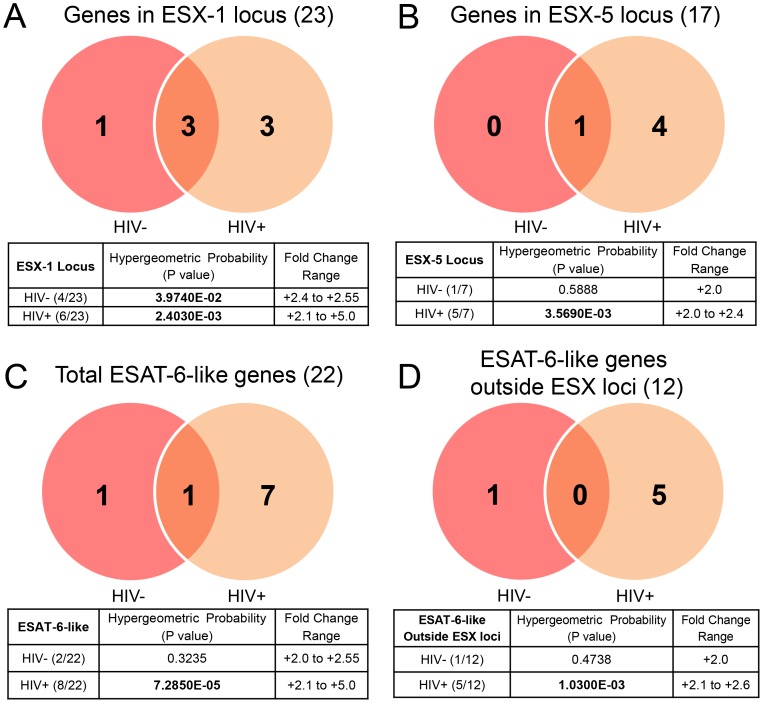
Upregulation of ESX loci and *esat-6*-like genes. (A) Genes upregulated within ESX-1 locus. (B) Genes upregulated within ESX-5 locus. (C) Total genes encoding ESAT-6-like proteins upregulated in the *M. tb* genome. (D) Genes encoding ESAT-6-like proteins which are upregulated and located outside of ESX loci. Venn diagrams indicate numbers of genes upregulated in blood from HIV- and/or HIV+ donors. Genes that are upregulated in both environments are in the overlap. Numbers in parentheses next to headings indicate the total number of genes in the designated category encoded in the *M. tb* genome. Tables below include the Hypergeometric Probability P-values from Fisher's Exact testing of differentially expressed genes represented in each category and each blood environment (HIV- or HIV+) (significant P-values in bold), as well as the ranges of expression fold change. Note: Genes located within ESX-2, ESX-3, and ESX-4 loci were not differentially expressed in blood from either HIV- or HIV+ donors.

*M. tb* utilizes a variety of secretion systems to export proteins across its cell-wall including the general (Sec) secretion system, the twin-arginine translocation (Tat) system, and multiple type VII secretion systems (T7SS) encoded within genomic loci termed ESX-1 to ESX-5 [79]. Only the expression of T7SSs encoded by ESX-1 and ESX-5 were extensively affected (Figure 6A,B). Genes within the ESX-1 locus were statistically represented among upregulated genes in both blood environments, while genes within the ESX-5 locus were statistically represented among upegulated *M. tb* genes in blood from HIV+ patients only (Figure 6A,B).

Rv3875 (*esat-6*), which encodes ESAT-6, an ESX-1 substrate, was upregulated in bacteria replicating in blood from both HIV- and HIV+ donors; in the latter environment, *esat-6* was the most highly upregulated gene (TABLE S3). Region of difference 1 (RD1), present in *M. tb* but absent from all BCG vaccine strains, encompasses the ESX-1 locus, and *M. tb* Δ*esat-6*, *M. tb* Δ*RD1* and BCG are similarly attenuated for cell-cell spread of *M. tb in vitro* and dissemination from the lungs to other organs *in vivo* [42], [80], [81]. ESAT-6 has been demonstrated to possess cytolytic activity for macrophages, alveolar epithelial cells, and red blood cells (RBC) and loss of ESX-1 or the ESAT-6 protein attenuates the cytolytic capability of *M. tb* [41], [42], [82]. Dormant *M. tb* down-regulate *esat-6* [83], and the presence of antibodies to ESAT-6 in retrospective sera obtained during the months prior to progression to clinical TB from HIV+TB+ patients confirms the *in vivo* expression of ESAT-6 during reactivation of latent TB [84]. In addition to *esat-6*, transcripts for ESX-1 locus-specific genes, Rv3864 (*espE*) and Rv3879c (*espK*), were upregulated in blood from both HIV- and HIV+ donors, and Rv3878 (*espJ*) was additionally upregulated in the HIV- donor blood. Rv3874 (*cfp-10*) which encodes the ESAT-6 binding partner, CFP-10, and two PE/PPE encoding genes within the ESX-1 locus, Rv3872 (*pe35*) and Rv3873 (*ppe68*) (known to be ESX-1 substrates in *M. marinum*) [85], [86], were upregulated only in HIV+ patient blood. The upregulation of ESX-1 T7SS and its substrate, ESAT-6; especially the expression of ESAT-6 in HIV+ patient blood, implicate a major role for this system in *M. tb* adaptation to the blood environment.

Several genes from the ESX-5 locus were also upregulated by *M. tb* replicating in blood from HIV+ patients (Figure 6B). Thus while Rv1787 (*ppe25*) was the only ESX-5 locus gene upregulated in blood from both HIV- and HIV+ donors, Rv1792 (*esxM*), Rv1798 (*eccA5*), Rv1790 (*ppe27*) and Rv1794 were upregulated in HIV+ patient blood exclusively. In addition, genes encoding three known substrates of the ESX-5 T7SS, located outside any ESX loci, were also upregulated- Rv2430c (*ppe41*) and Rv0442c (*ppe10*) in bacteria replicating in blood from both HIV- and HIV+ donors and Rv2431c (*pe25*) in HIV+ patient blood alone. ESX-5 plays an important role in the secretion of PE/PPE proteins [87], [88], and the upregulation of ESX-5 is consistent with the observation that the family of PE/PPE genes is the second most upregulated functional category in *M. tb* replicating in the blood (TABLE 1 and Figure 4A). ESX-5 function has been linked to *M. tb* cell-wall integrity and virulence [88]. Importantly, deletion of a region encompassing five genes in the ESX-5 locus, Rv1787-Rv1791 (*ppe25-pe19*), attenuated *M. tb* growth in lungs and spleen of intravenously infected SCID mice [88], suggesting a role for ESX-5 in the hematogenous dissemination of *M. tb.* Coincidently, *ppe25* and *ppe27*, located in the above described deleted region of ESX-5, were upregulated in the HIV+ patient blood.

ESAT-6 belongs to a family of proteins called “ESAT-6-like” proteins, characterized by their small size (∼100 amino acids), helix-turn-helix structure, centrally located WXG motif, and ability to be secreted despite the lack of a classical secretion signal [89]. In total, *M. tb* encodes 22 ESAT-6-like proteins. Genes for 2/22 ESAT-6-like proteins were upregulated during growth in blood from HIV- donors; 8/22 were upregulated in HIV+ patient blood making this group of genes significantly represented in the latter environment (Figure 6C). Genes for ten of the 22 “ESAT-6-like” genes, including ESAT-6 and CFP-10 (also ESAT-6-like), are located in tandem pairs within ESX loci, and the remainder are located elsewhere in the genome (Figure 6D). Interestingly, transcripts for 6 of the 12 ESAT-6-like proteins that are encoded outside the ESX loci were upregulated in *M. tb* replicating in blood; 1 in blood from HIV- donors and 5 in blood from HIV+ patients, giving statistical significance to the upregulation of this group of genes in the HIV+ blood environment (Figure 6D). Thus, while Rv3950c (*esxF*) is upregulated bacteria replicating in blood from HIV- donors, Rv1038c (*esxJ*), Rv1197 (*esxK*), Rv1198 (*esxL*), Rv2347c (*esxP*), and Rv3620c (*esxW*), were upregulated exclusively during replication in the HIV+ patient blood. It is not known if the secretion of these ESAT-6-like proteins occurs via the ESX-1 or ESX-5 secretion systems. Also, while ESAT-6 and CFP-10 have been studied extensively, the functions of the other ESAT-6-like proteins remain to be investigated. (A categorized listing of all upregulated ESX- and ESAT-6- related genes is provided in TABLE S4.)

### Iron acquisition by *M. tb* in whole blood

The mechanisms for acquiring iron during iron scarcity and storing iron during excess are carefully orchestrated in *M. tb* in response to the availability of iron [90]. No genes induced in low-iron conditions were upregulated during growth in the blood environment when compared to *M. tb* growing in Middlebrook 7H9 media which is high in free iron. In contrast, transcripts for two iron-repressed genes, Rv1169c and Rv1463, were down-regulated in blood from both HIV- and HIV+ donors. Rv2711, the gene encoding IdeR, a transcriptional regulatory protein which functions as an iron-binding repressor of siderophore biosynthesis and iron uptake, was also upregulated in blood from HIV- donors. Furthermore, the IdeR- and iron-induced gene Rv3841, encoding the bacterioferritin BfrB, was upregulated by *M. tb* in the HIV+ patient blood. The function of BfrB is to store iron when it is in excess to avoid iron toxicity and to make it available during times of iron scarcity [91]. Interestingly, BfrB elicits antibody responses in both HIV- TB and HIV+ TB patients, indicating that a high free iron environment is experienced by *M. tb* during human infection [92], [93]. Together these transcriptional alterations indicate adequate supply of iron in the *M. tb*- infected blood environment. These results were unexpected since access to free iron in the host is typically limited by high-affinity iron chelators such as transferrin in the blood and ferritin within host cells [94], and *M. tb* siderophores are known to acquire iron from transferrin [95], suggesting that alternative mechanisms likely contribute to iron acquisition in the blood environment.

Another mechanism by which iron can become available to pathogens in the blood is through hemolysis [94] . Mechanisms for iron acquisition from heme and heme-related molecules are known in other bacteria [96], [97], and *M. tb* has been shown to be capable of acquiring iron from heme by a siderophore-independent mechanism; however, genes responsible for *M. tb* import of heme iron (Rv0202c-Rv0207c) and the gene encoding the secreted heme-binding Rv0203 were also not affected in the blood environment [98], [99], [100]. *esat 6* is highly upregulated in the HIV+ patient blood, and ESAT-6 has been demonstrated to lyse RBCs [82]. It is tempting to speculate that the hemolytic activity of ESAT-6 might contribute to providing a rich source of iron to *M. tb* in the blood of HIV+ patients by releasing iron-bound hemoglobin from RBCs.

Such hemolytic activity would lead to anemia, and in fact, anemia is a common complication of TB [101]. While iron deficiency, anemia of inflammation, and hemoptysis are all contributors to TB-associated anemia [102], hemolysis due to the activity of ESAT-6 (and possibly other ESAT-6-like proteins), could potentially also contribute to driving anemia in HIV+ TB patients with bacteremia. Interestingly, TB-associated hemolytic anemia is resolved by successful treatment with ATT in the absence of transfusions, corticosteroids, or iron supplementation indicating that TB alone was the cause of anemia [103]. Furthermore, anemia and HIV seropositivity have been identified as strong clinical indicators of mycobacteremia [104], [105]. While *M. tb* bacteremia can occur in both HIV- TB and HIV+ TB patients [106], [107], it is far more common in the latter cases [24], [108]. Host sequestration of iron in the blood is an important mechanism against bacteremia; however, due to the release of iron, persons with hemolytic disorders are more susceptible to bacteremia with pathogens such as *Salmonella* and *Pneumococcus*, [94], [109]. The potential role of ESAT-6 in *M. tb* iron acquisition during hematogenous dissemination and TB-associated anemia in HIV+ patients remains to be investigated.

### Quantitative real-time polymerase chain reaction (qRT-PCR) validation of microarray results

**Figure 7:**
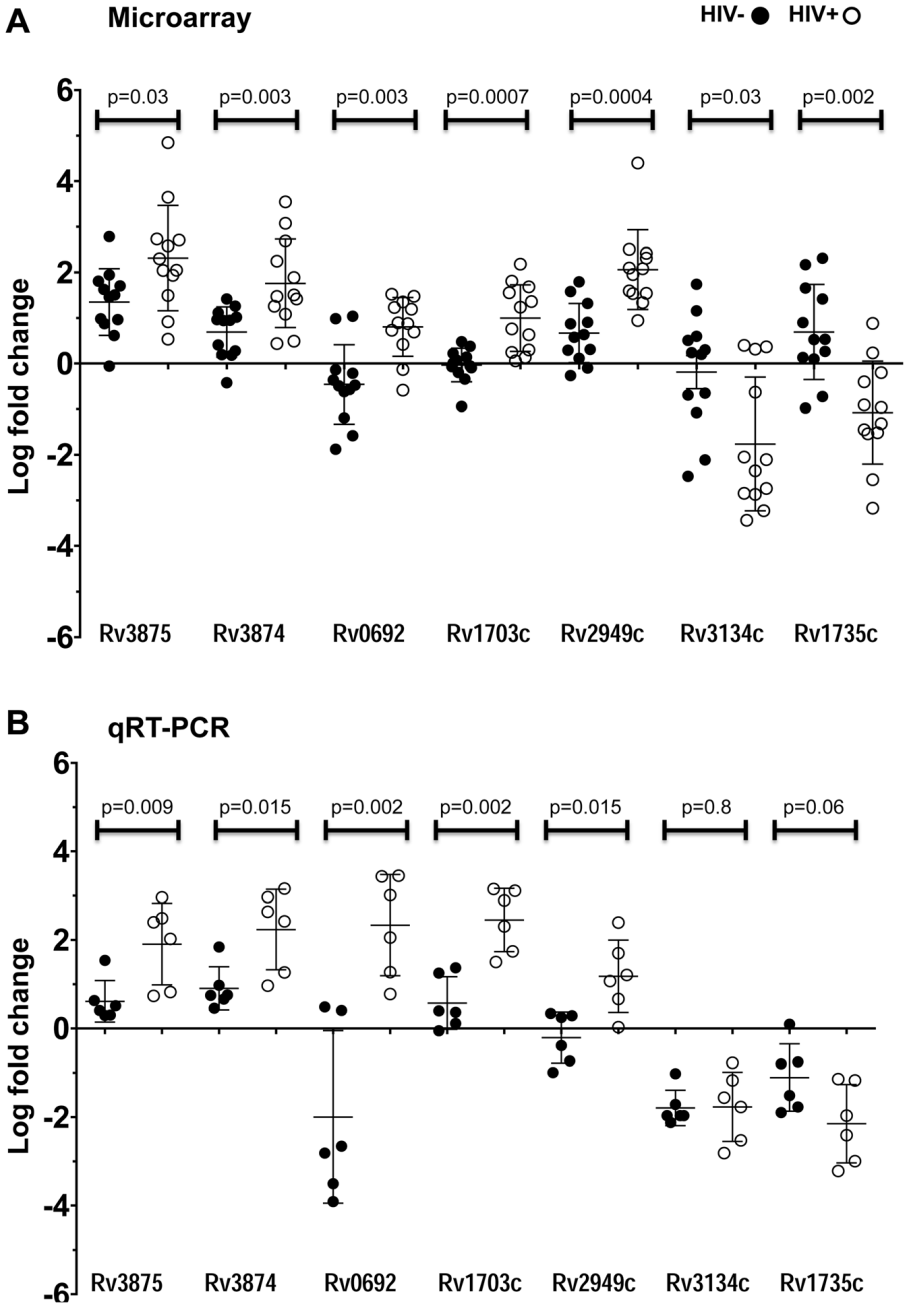
qRT-PCR validation of microarray results. Log_2_ fold-changes in expression of seven selected genes in *M. tb* replicating in blood from HIV- donors (black dots) or from HIV+ patients (open black circles) at 96 hr compared to *M. tb* logarithmically growing in 7H9 broth media. (A) Microarray results from 6 biological replicates (6 different donors) per condition (blood from HIV- or HIV+ donors) with 2 technical replicates (Cy5/Cy3 dye swap) (total 12 values per condition are shown). (B) qRT-PCR results from *M. tb* replicating in blood from 6 different donors per condition (HIV- or HIV+) (total 6 values per condition are shown). Y-axis values (Log_2_ fold change) of ≥1 indicate upregulation and values ≤-1 indicate down-regulation. (For qRT-PCR, numbers of transcripts were normalized to copies of 16S rRNA.) P-values indicate the statistical difference between the average log_2_ fold-change of a gene in blood from HIV- subjects compared to HIV+ patient blood by Mann-Whitney two-tailed test.

Seven *M. tb* genes representing different functions and varying levels of transcriptional regulation in blood from HIV- and HIV+ donors were selected for verification of the microarray results by qRT-PCR (Figure 7). In contrast to the microarrays, the qRT-PCR was performed on unamplified *M. tb* RNA. Genes chosen for the qRT-PCR analysis included ESX-1 genes Rv3875 (*esat-6*), Rv3874 (*cfp-10*), as well as DevR (DosR)-regulated genes Rv3134c, encoding a universal stress protein, and Rv1735c, of unknown function. Randomly selected genes included Rv0692, Rv1703c and Rv2949c. In *M. tb* replicating in HIV+ patient blood, the four genes that were upregulated in the microarrays (*esat-6*, *cfp-10*, Rv1703c and Rv2949c) were also upregulated in the qRT-PCR analysis, and the two selected genes down-regulated during growth in HIV+ patient blood (Rv3134c and Rv1735c) were also down-regulated in the qRT-PCR analysis. In comparison, all of the selected genes, with the exception of *esat-6*, were not differentially expressed in blood form HIV- donors in the microarray study. Of these, four genes (*cfp-10*, Rv1703c, Rv2949c, and Rv1735c) were also not differentially expressed by qRT-PCR analysis. In blood from the HIV- donors, *esat-6* was upregulated on the microarrays; but was not statistically significantly differentially expressed by qRT-PCR, although the results trended towards a positive fold change. Similarly, while Rv0692 was down-regulated in blood from HIV- donors by qRT-PCR, the differential expression on the microarrays only trended towards a negative fold change. This same gene was upregulated in blood from HIV+ patients by qRT-PCR but only trended towards upregulation by microarray. Rv3134c was down-regulated in HIV+ patient blood by both assays and in blood from HIV- donors by qRT-PCR but was not identified to be differentially expressed by microarray. Therefore, Rv3134c (in blood from HIV- donors) was the only gene whose expression did not mirror or trend similarly in both assays. For five *M. tb* genes, the statistically significant difference between expression in blood from HIV- donors versus HIV+ patients coincided in both microarray and qRT-PCR analysis.

### Comparison of *M. tb* transcriptional profile during replication in blood and in macrophages

The transcriptional profile of *M. tb* in the intra-macrophage environment has been investigated extensively [37], [38], [110]. A comparison of the key transcriptional adaptations of *M. tb* in the macrophage (human differentiated monocytic cell-line THP-1 (THP-1) and activated murine bone marrow-derived macrophages (mBMM) [37], [110]), and in the blood reveals distinct responses to the two environments (TABLE 2). While expression of some genes differ in the two intra-macrophage environments, a number of *M. tb* genes were upregulated in both THP-1 and mBMM; including Rv0080, Rv0081, Rv1169c (*pe11*), Rv2028c, Rv2029c (*pfkB*), Rv2030c, Rv2031c (*hspX*), Rv2032 (*acg*), Rv2429 (*ahpD*), Rv2430c (*ppe41*), Rv2626c, Rv2930 (*fadD26*), Rv3133c, Rv3139 (*fadE24*), and Rv3140 (*fadE23*) [37], [110]. Of these, transcripts for Rv2930 (*fadD26*) and Rv2429 (*ahpD*) were also upregulated in blood from both HIV- and HIV+ donors. Interestingly, Rv2430c (*ppe41*), upregulated in both blood and intra-macrophage environments, is an immunodominant B-cell inducer in patients with active TB, primary and reactivated, as well as EPTB [111], and is a substrate of the ESX-5 T7SS [88]. In contrast, Rv2031c (*hspX*) and Rv1169c (*pe11*), were down-regulated in blood from both HIV- and HIV+ donors, and Rv2029c (*pfkB*), and Rv2030c were additionally down-regulated in HIV+ patient blood. The remaining *M. tb* genes commonly upregulated in THP-1 and mBMM were not differentially expressed by *M. tb* in the blood. Among *M. tb* genes upregulated in the THP-1, but not affected in mBMM, is the *mymA* operon (Rv3083-Rv3089). Rv3083 was down-regulated in blood from both HIV- and HIV+ donors. Rv3088 and Rv3089 (*fadD13*) were additionally down-regulated in the HIV+ patient blood. Rv3742c, a gene of unknown function, upregulated in THP-1 but not affected in mBMM, was also down-regulated exclusively in the HIV+ patient blood. Importantly, *esat-6,* upregulated during replication in blood from both HIV- and HIV+ donors, most strongly in the latter, was unaffected in THP-1 and down-regulated in mBMM.

**Table 2:** Comparison of *M. tb* genes differentially expressed in HIV- donor and/or HIV+ patient whole blood with corresponding *M. tb* genes in the intra-macrophage environment from two published studies.

Gene(s)	HIV- blood^a^	HIV+ blood^a^	THP-1^b^	mBMM (activated)^c^
Rv3875 (*esat-6*)	Up	Up	NDE	Down
Rv3083 (*mymA* operon)	Down	Down	Up	NDE
Rv3084 (*mymA* operon)	NDE	NDE	Up	NDE
Rv3085 (*mymA* operon)	NDE	NDE	Up	NDE
Rv3086 (*mymA* operon)	NDE	NDE	Up	NDE
Rv3087 (*mymA* operon)	NDE	NDE	Up	NDE
Rv3088 (*mymA* operon)	NDE	Down	Up	NDE
Rv3089 (*mymA* operon)	NDE	Down	Up	NDE
Rv2029c (*pfkB*)*	NDE	Down	Up	Up
Rv2030c*	NDE	Down	Up	Up
Rv2031c (*hspX*)*	Down	Down	Up	Up
Rv3873 (*ppe68*)	NDE	Up	Down	Down
Rv3742c	NDE	Down	Up	NDE
Rv3841 (*bfrB*)	NDE	Up	Down	Down
Rv1169c (*lipX*, *pe11*)	Down	Down	Up	Up
Rv2930 (*fadD26*)	Up	Up	Up	Up
Rv2429 (*ahpD*)	Up	Up	Up	Up
Rv2430c	Up	Up	Up	Up

a This study.

b human differentiated THP-1cell line (THP-1)- (Fontan P et al., 2008) (110).

c activated murine bone marrow derived macrophages (mBMM)- (Schnappinger D et al., 2003) (37).

*Belongs to DevR (DosR) regulon.

“Up” indicates upregulated, “Down” indicates down-regulated, “NDE” indicates “not differentially expressed”. Note: The genes listed in this table are key to the studies cited. This TABLE is a representative but not exhaustive list.

The intra-macrophage is a low iron environment [60], thus Rv3841 (*bfrB*), was down-regulated in both THP-1 and mBMM; as mentioned above *bfrB* was upregulated in the HIV+ patient blood. Also down-regulated in THP-1 and mBMM, Rv3873 (*ppe68*) of the ESX-1 locus is upregulated in the HIV+ patient blood. DevR (DosR) regulon transcriptional responses in blood and macrophages were conspicuously different; 3/48 and 21/48 genes were down-regulated during replication in blood from HIV- and HIV+ donors, respectively (Figure 5A), while none were down-regulated in THP-1 or mBMM, and in fact, 9 DevR (DosR) regulon genes were upregulated in both THP-1 and mBMM and an additional 32 were upregulated in the latter [37], [110] (TABLE S5 and Figure S5). The distinct transcriptional changes adopted by *M. tb* replicating in the blood versus the macrophage indicates that during hematogenous phase of infection, macrophages are not an exclusive niche for *M. tb*.

### ESAT-6 stimulates HIV replication in U1 cells

**Figure 8:**
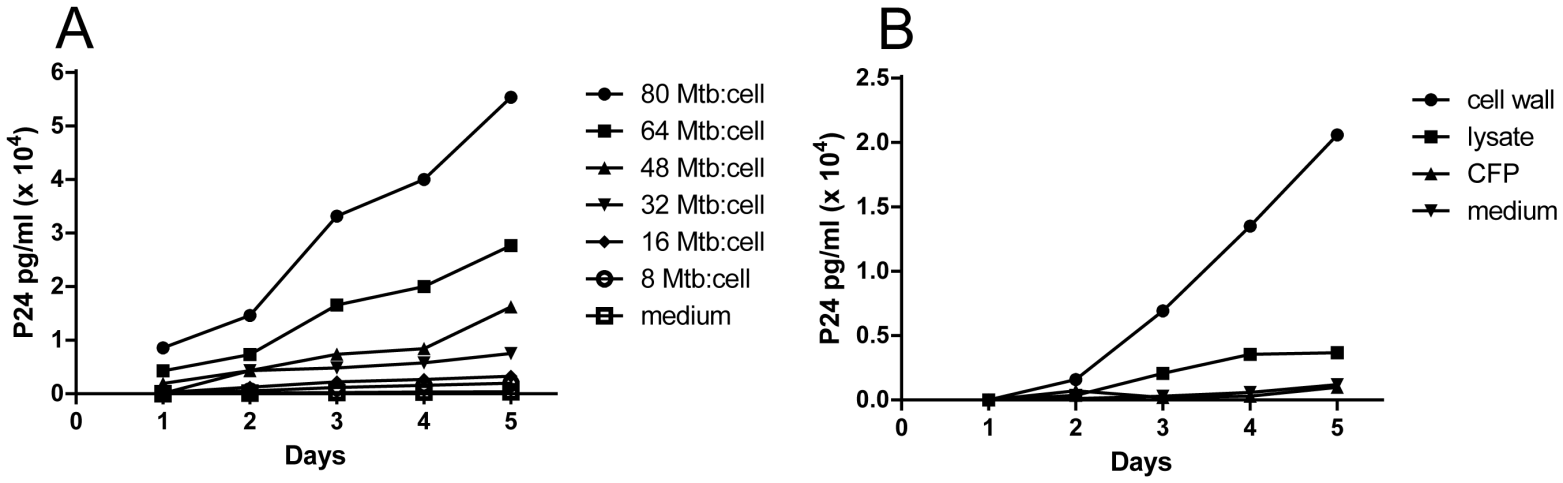
Effect of *M. tb* on HIV-1 p24 production by U1 cells. (A) Effect of gamma-irradiated, intact *M. tb* on p24 production by U1 cells over 5 days at increasing MOI (*M. tb*:cell). (B) Effect of *M. tb* subcellular fractions- cell wall, lysate, and culture filtrate proteins (CFP) at 20 ug/ml on U1 p24 production over 5 days. U1 culture medium alone represents the negative control in both assays. Shown are representatives of two or more experiments.

*M. tb*:HIV co-infection creates a synergistic environment which drives and accelerates both infections [112], [113]. HIV viral load during the acute phase of TB is reported to increase 5–160 fold in plasma [114], and viral burden in bronchoalveolar lavage fluid (BAL) from TB-involved lung is higher than in BAL from uninvolved lung of TB+ HIV+ patients or from TB- HIV+ patients indicating a local effect of *M. tb* on viral burden [115]. Additionally, blood monocytes from patients with active TB are more susceptible to productive HIV-infection [116], and *M. tb* PPD induces HIV replication in the U1 cell line, a model of HIV latency containing HIV-1 proviruses [117]. Cell culture supernatants from U1 cells incubated with intact gamma-irradiated *M. tb* H37Rv exhibited p24 production which increased in a *M. tb* dose-dependent manner (Figure 8A). When U1 cells were stimulated with subcellular fractions of *M. tb*, the cell-wall fraction dramatically induced HIV replication while neither cell lysate nor culture filtrate protein fractions induced appreciable p24 production (Fig 8B). Purified recombinant ESAT-6 and two other cell-wall proteins, CFP-10 and malate synthase (MS, Rv1837c), were tested for their ability to stimulate HIV replication in U1 cells (TABLE 3); only ESAT-6 induced HIV replication (>100 fold at 2 ug/ml; a concentration not cytotoxic to U1 cells, data not shown). Neither CFP10 nor MS induced significant HIV replication even at concentrations as high as 10 ug/ml (data not shown). Preliminary experiments with similar concentrations of the surface-exposed and secreted antigens ERP, MPT51, and Ag85c (Rv3810, Rv3803c, and Rv0129c, respectively) also failed to induce p24 production by U1 cells (data not shown). ESAT-6 is present in the cell-wall, cell-membrane and the culture filtrates of *M. tb* [118], [119], [120], [121]. The inability of the culture filtrate fraction to induce p24 production in our experiments (Figure 8B) is likely because the CSU/BEI culture filtrate protein preparation method excludes proteins <10 kDa [122], [123] and has little, if any ESAT-6 (∼6 kDa). These results specifically implicate ESAT-6 in the synergistic interplay between *M. tb* and HIV during co-infection, with an HIV+ environment stimulating the transcription of *esat-6* and thus perhaps increasing *M. tb* virulence, and *M. tb* ESAT-6, in turn, stimulating HIV replication in HIV-infected macrophages. The mechanism(s) by which ESAT-6 drives HIV replication merits further investigation.

**Table 3:** Fold increase in U1 p24 production induced by *M. tb* recombinant proteins.

Protein	0.5 ug/ml	1 ug/ml	2 ug/ml
ESAT-6	1.93+/−0.69	20.3+/−0.84	111.65+/−35.7
CFP-10	0.69+/−0.084	1.06+/−0.61	0.44+/−0.40
MS	1.41+/−0.01	1.53+/−0.007	1.92+/−1.0

Numbers indicate the fold change in p24 levels in supernatants of protein-treated cells at day 5 of incubation compared to p24 levels produced by U1 cells in wells with medium alone. Means and standard deviations from two independent experiments are shown.

### Concluding Remarks

Hematogenous dissemination of *M. tb* occurs during the natural course of establishment of latent infection leading to the systemic seeding of the host organs [1], [2], [3], [4], [5], [8]. Hematogenous presence of *M. tb* is also frequently observed during both primary and reactivation pulmonary and extrapulmonary TB, and in disseminated and miliary TB. The ability of *M. tb* to adapt to the physiological and immunological microenvironments it encounters enables avoidance of the host anti-bacterial mechanisms and transition between latent and active states. While the adaptation to important environments such as the lungs, macrophages and the granuloma have been investigated, scant information is available regarding *M. tb* adaptation to the hematogenous environment. HIV+ patients are at a significantly enhanced risk for active TB infection and progression, for manifestation of extrapulmonary and systemic forms of TB [10], [16], [17], [18], [19], [20], and for *M. tb* bacteremia [24], [25], [108], [124]. The enhanced risk for TB arises independently of CD4+ T cell numbers, viral load or antiretroviral treatment, although the degree of risk correlates with immune dysfunction [11], [12], [13], [15], [125], [126]. Interestingly, studies with HIV+ TB patients prior to and through the institution of Highly Active Antiretroviral Therapy (HAART) showed that HAART decreases the risk for pulmonary TB more than the risk for EPTB [127], suggesting that treatment may have lower ability to attenuate systemic dissemination. These cumulative observations suggest that during residence in blood, *M. tb* itself alters its phenotype to drive active TB, and more so in the blood from HIV+ patients.

To our knowledge, this is the first investigation that specifically delineates the “blood specific transcriptome” of *M. tb* and provides insight into the bacterial mechanisms that likely drive active TB and disseminated forms of TB. Thus, *M. tb* is capable of replicating in and adapting to the blood of both HIV- healthy donors and HIV+ patients (Figures 1 and 2). *M. tb* in the blood environment undergoes a transcriptional adaptation indicative of a more aggressive phenotype as demonstrated by the adaptation to an active rather than dormant state, potential dampening of immune recognition by down-regulating the heat shock response, cell-wall remodeling consistent with increased virulence, and upregulation of ESX-1 and ESX-5 secretion systems and substrates associated with virulence. Interestingly, the numbers of genes affected in each of these categories were higher in *M. tb* replicating in blood from HIV+ patients than in blood from the HIV- donors (Figures 5 and 6). We interpret this effect to be attributable to *M. tb*-adaptive rather than HIV-driven mechanisms since the viral loads in nearly all HIV+ blood patients were undetectable and the CD4+ T cell counts in four of the six patients whose blood was used in the microarray analysis were in the normal range (). Importantly, the HIV+ patients included in this study were all receiving HAART. It is likely that in blood from HIV+ HAART-naïve individuals the *M. tb* transcriptional adaptations might be even more striking, and these studies are planned.

Another striking finding in this study was the dramatic upregulation of the gene encoding the virulence factor, ESAT-6 during *M. tb* replication in both blood environments; *esat6*, was the most upregulated of all genes in *M. tb* growing in blood from HIV+ patients (Table S3). ESAT-6 is a cytolytic virulence factor which participates in the bacterial crossing of the alveolar barrier [41], and also has hemolytic activity that may facilitate *M. tb* access to iron during the hematogenous phase of infection. Our preliminary studies with recombinant purified ESAT-6 suggest that it may also play a specific role in enhancing HIV replication in the infected macrophages thus contributing to the accelerated progression of HIV-infection to AIDS.

The whole blood model has been used to study the transcriptional adaptation of other human bacterial pathogens during bacteremia [27], [28], [29], [30]. In contrast to studies with isolated cell-types, the whole blood represents a complete environmental context encountered *in vivo* during hematogenous phases of infection. *M. tb*-infected neutrophils produce cytokines that can activate macrophages, and dendritic cells, NK cells, and γδ T cells are involved in the innate immune responses to *M. tb* [128], [129], [130], [131], [132]. Thus, the macrophage model may provide insufficient insight into the *M. tb* mechanisms that support infection and disease progression. The whole blood model includes all the cell types that participate in immune responses, including macrophages, different T cell populations (CD4+, CD8+, TH17, T regs), dendritic cells, monocytes, neutrophils (and other granulocytes) etc as well as the resulting complex milieu of cytokines and chemokines [133], [134]. In fact, circulating cytokine levels and other biomarkers have been shown to correlate with TB disease and response to treatment [135], [136], and the whole blood model has been used to assay the ability of drugs to kill *M. tb* [137]. The comparison of the significant findings in this study with those from published microarray studies of *M. tb* within macrophages [37], [110] highlights the differential responses of *M. tb* in the whole blood environment and within isolated cells (Table 2).

The ability of *M. tb* to grow in whole blood *ex vivo* has been suggested to correlate with virulence of the infecting *M. tb* strain [138]. Our results suggest that *M. tb* alters its phenotype in the hematogenous environment and adapts transcriptionally by enhancing its virulence potential. Our studies also demonstrate that *M. tb* can distinguish between the blood environments of healthy, immunocompetent donors and of HIV+ patients. While the current studies provide a snapshot of the transcriptional adaptation in blood at a single time point in blood from HAART-treated HIV+ patients, it is likely that *M. tb* adapts to the continuously changing immunological environment in HIV+ patients to exploit the optimal opportunities for its growth, and time spent in the hematogenous environment may also affect the degree of adaptation. The results of this study will drive future investigations aimed at understanding the pathogenesis of *M. tb* and in the identification of diagnostic biomarkers and therapeutic targets for active TB, *M. tb* bacteremia, and TB in HIV+ individuals.

## Materials and Methods

### Ethics Statement

Peripheral whole blood was collected from PPD-, healthy, HIV- volunteers working at the Veterans Affairs Medical Center (VAMC) with written informed consent signed by the participants (NYU School of Medicine-IRB approved study #07-848; Board A (IRB#1), FWA 00004952. Whole blood from de-identified HIV+ patients that otherwise would have been discarded after analysis for CD4+ T cell counts and viral load assessment (NYUSoM, IRB approved study #7279; Board B (IRB#2), FWA 00004952) was also used in this study.

### Human Subjects

Peripheral blood from 13 PPD-negative, HIV- healthy volunteers who had not received BCG vaccination and 17 HIV+ patients on HAART undergoing routine monitoring for viral loads and CD4+T cells were obtained. The PPD status of the HIV+ donors was presumed to be PPD-negative since all were US born. The details of CD4+ T cell count and viral load for each HIV+ patient included in these studies (CFU, Microarray, or qRT-PCR) are listed in TABLE S1.

### Whole blood *M. tb* culture

*M. tb* H37Rv was cultured in whole blood as described [139]. Briefly, whole blood was diluted 1∶1 with RPMI-1640 supplemented with 1% L-glutamine and 20 IU/ml heparin (Sigma). Six aliquots of diluted blood (0.9 ml) from each subject were inoculated with 0.1 ml (10^6^ CFU) of single cell suspensions of log phase *M. tb* H37Rv grown in Middlebrook 7H9 media in 7 ml endotoxin-free Sterilin non-pyrogenic tubes (Dynalab corporation) and incubated at 37°C shaking at 20 rpm. For CFU determination, at 2 hr and 96 hr, three inoculated blood samples per subject were treated with 0.1% triton X 100 (Sigma) in water containing 6M urea (Sigma) for 10 min at room temperature to lyse blood cells. Lysed infected blood samples were centrifuged at 2000×g for 15 min to collect the bacteria, and the pellets were washed once with the same buffer and resuspended in MiddleBrook 7H9 media with 0.05% Tween 80. For CFU determination, serial dilutions of these suspensions were plated onto MiddleBrook 7H11 agar plates and incubated at 37°C. After 14-21 days CFU were counted. CFU counts from triplicate infections per time point were averaged.

### Isolation of *M. tb* from blood cultures and extraction of *M. tb* RNA

Ten-20 ml diluted whole blood was inoculated with *M. tb* H37Rv (as described above) in 100 or 150 ml Sterilin endotoxin-free containers (Dynalab corporation) and incubated at 37°C. At 96 hr post inoculation, blood cells were lysed, the bacteria pelleted by centrifugation and the bacterial pellets were immediately resuspended in GTC solution (4 M guanidium thiocynate, 0.5% sodium N-lauryl sarcosine, 25 mM tri-sodium citrate pH 7.0, 0.1 M 2-mercaptoethanol and 0.5% tween 80) to lyse any remaining eukaryotic cells. After washing once each with GTC solution and 0.5% tween 80, the bacterial pellets were each resuspended in 1 ml TRI reagent containing polyacrylamide carrier (Molecular Research Center). For RNA isolation, the bacilli were disrupted in a bead beater with autoclave-sterilized 0.1 mm zirconia beads (Biospec) and resulting lysates used to extract total RNA as described [140]. The RNA preparations were cleaned by RNeasy columns (Qiagen) after treatment with Turbo DNase (Ambion). RNA quality and quantity was determined using an Agilent 2100 Bioanalyzer.

Log phase *M. tb* grown in 7H9 broth were treated similarly to obtain reference RNA. Ten ml Middlebrook 7H9 media was inoculated with a 0.5 ml frozen aliquot of bacteria (OD600 = 0.7 at time of storage) yielding a starting culture of OD600 ∼0.035. The culture was incubated at 37°C with shaking (110 rpm) for 7–9 days and harvested at an OD600 of 0.7–0.9 (mid to late log phase). Bacteria similarly cultured enter stationary phase at OD600 ∼1.4 to 1.7.

### DNA Microarrays

RNA from *M. tb* cultured in blood from 6 PPD- HIV- healthy donors and 6 HIV+ patients was used for the DNA microarray studies with RNA from *M. tb* grown to log phase in Middlebrook 7H9 broth as the reference (see TABLE S1 for HIV+ donor details). To ensure adequate amounts of RNA for the microarray study, an RNA amplification strategy was used on similar amounts of all *M. tb* RNA samples [141], [142]. Briefly, each RNA sample (100 ng) was amplified using MessageAmp II-Bacteria RNA amplification kit (Ambion). Since this method produces aRNA in antisense orientation, the synthesis of cDNA from aRNA and fluorescent labeling of cDNA with Cy3 or Cy5 was accomplished by first performing reverse transcriptase (RT) reaction with Superscript II RT followed by labeling with Klenow fragment using Bioprime Kit (Invitrogen) following a published method [141]. This amplification method has been used to study differential gene expression in several organisms [141], including *M. tb* obtained from sputum samples of TB patients [142]. We first confirmed that the expression profiles of amplified *M. tb* RNA (aRNA) are similar to those of unamplified RNA, and the results are consistent with earlier reported studies [143] (Figure S6). The *M. tb* microarray chips containing 70-mer oligonucleotides representing all open reading frames annotated in the *M. tb* H37Rv genome sequence were obtained from the Center for Applied Genomics (Public Health Research Institute; Newark, NJ). Labeled cDNA from aRNA of log phase *M. tb* grown in 7H9 broth was used as the reference control on each chip. For hybridization, labeled cDNA probes generated from aRNA obtained from *M. tb* grown in each blood sample was mixed with the labeled reference cDNA probe prior to purification with Microcon YM10 filter (Millipore). For each *M. tb*-infected blood sample, the *M. tb* arrays were hybridized over night with the mixed labeled cDNA probes in duplicate to include a Cy3/Cy5 dye swap [110]. The microarrays were scanned and processed with an Axon 4000B scanner and GenePix Pro 6.1 software, respectively. The chips were normalized by the print-tip Lowess method, and the Cy5/Cy3 intensity ratio was determined for each gene [144]. The intensity ratio data obtained from *M. tb* grown in blood from all subjects in the HIV- or HIV+ group was used to perform one class Significance Analysis of Microarrays (SAM) with Multiarray Viewer Software on the TMEV website for determination of differentially expressed genes of *M. tb* grown in whole blood compared to 7H9 broth grown *M. tb* [145]. Among genes identified by SAM, only genes differentially expressed in the blood environments compared to 7H9 broth that showed a ≥2 fold change at a false discovery rate of <2% were considered significantly differentially expressed. Data were deposited in Gene Expression Omnibus repository (Accession Number: GSE49760). To contrast the *M. tb* differential gene expression in the two blood groups (HIV+ vs HIV-) (Figure 3), HIV+/7H9 log ratios and HIV-/7H9 log ratios (data obtained above) were directly compared by two class unpaired SAM analysis, and a ≥2 fold difference (HIV+ vs HIV-) at a false discovery rate of <2% were considered significantly differentially expressed.

### Statistical Analysis

Hierarchical clustering of total upregulated and total down-regulated genes was performed using TMEV MeV_4_9_0 software with the distance threshold set at 0.75. Fisher's Exact Test (Microsoft Fisher's Exact Test Calculator) was applied to the data to determine the hypergeometric probability of enrichment amongst functional catergories of interest.

### Quantitative RT-PCR

To validate the differential expression of *M. tb* genes identified in the microarray, expression of seven selected genes was determined in unamplified RNA from *M. tb* grown in blood from 6 HIV- and 6 HIV+ subjects (TABLE S1) by qRT-PCR [41]. *M. tb* gene specific primers were designed using Primer3 software (TABLE S6). Briefly, *M. tb* RNA was subjected to synthesis of first-strand cDNA using Superscript II RNase H^-^ reverse transcriptase (RT). The real-time PCR was performed using iQ SYBR Green supermix, primers, and cDNA in a MyiQ2 two color Real Time PCR Detection System (Bio-Rad). For each RNA, a reaction without RT was performed as a negative control. For quantitation, a standard curve was generated for each gene using a serial dilution of *M. tb* genomic DNA and respective primers. Copies of 16S rRNA was used to normalize the transcript levels of the respective genes. The ratio of normalized copies of each selected gene from *M. tb* grown in blood from HIV- or HIV+ subjects to normalized copies in 7H9 broth-grown *M. tb* was calculated to determine the fold change in expression of that gene in HIV- or HIV+ blood compared to reference 7H9 broth. Prior to quantitation, the specificities of PCR products were verified by amplification of each gene target using respective primers with *M. tb* H37Rv genomic DNA and cDNA obtained from *M. tb* RNA isolated from infected blood as templates and sequencing of amplified products. Mann-Whitney two-tailed test using GraphPad prism software was used for statistical analysis, and P-value<0.05 was considered statistically significant.

### Culture of U1 cells

U1 cell line which is a chronically infected clone from the parent promonocyte cell line, U937, and contains two HIV-1 pro-viruses integrated into the genome, was obtained from the NIH AIDS Research and Reference Reagent Program (Rockville, MD). The U1 cells were grown in RPMI 1640 medium (Cambrex, MD) supplemented with 10% of fetal calf serum, 2 mM L-glutamine and 1% penicillin /streptomycin at 37°C in a 5% CO_2_ incubator. Cells were diluted to 2×10^5^/ml and passaged every 5 days. Low level of p24 (∼100–400 pg/ml) was detected at day 5 from the supernatant of untreated U1 cells. Cell viability was monitored by trypan blue exclusion and more than 95% cells were viable on day 5.

### Treatment of U1 cells with γ-irradiated *M. tb*

U1 cells were washed twice with RPMI. One ml of U1 cell suspension (2×10^5^ cells) was suspended in wells of a 24-well plate and duplicate wells incubated with γ-irradiated *M. tb* H37Rv at multiplicities of infection (MOI) (bacteria:cell) ranging from 8∶1 to 80∶1. The plates were incubated at 37°C in a 5% CO_2_ incubator, and 100 ul/well supernatant removed daily for 5 days and replaced by 100 ul of fresh medium to maintain constant volume. The collected viral supernatants were mixed with 100 ul of PBS with 0.05% Tween 20 (PBST) containing 2% Triton X 100 and frozen until tested by p24 ELISA described below.

### Treatment of U1 cells with *M. tb* subcellular fractions

Subcellular fractions of H37Rv (total cell lysate, cell-wall, and culture filtrate proteins (CFP) were obtained from the NIH/NIAID TB Research Materials and Vaccine Testing Contract, Colorado State University (CSU), Fort Collins (currently provided by (BEI Resources: www.beiresources.org) and were incubated in duplicate with U1 cells (2×10^5^ cells in 1 ml) at final concentrations of 4 ug/ml, 20 ug/ml, and 100 ug/ml in cell culture medium at 37°C in 5% CO_2_ for five days (representative data with cell fraction concentrations of 20 ug/ml is shown). Incubation of cells with cell culture medium alone was used as a negative control. Cells were allowed to settle and 100 ul supernatants of treated and untreated cells were collected daily for 5 days and subjected to p24 ELISA as described below.

### Treatment of U1 cells with purified *M. tb* proteins

Purified *M. tb* proteins (ESAT-6, CFP-10, and malate synthase) were also obtained from the above source at CSU. U1 cells were incubated with increasing concentrations (0, 0.5, 1, and 2 µg/ml) of the 3 purified proteins for 5 days at 37°C in 5% CO_2_. On day 5, cells were allowed to settle and 100 ul supernatants of treated and untreated cells were collected. P24 was measured by ELISA. Fold increase in p24 production was determined by dividing the amount of p24 detected in the supernatant of protein-treated cells with the amount of p24 detected in the supernatant of untreated cells on day 5 of incubation.

### Measurement of p24 production by U1 cells

The concentration of p24 in the supernatants of U1 cell cultures was measured by an in-house ELISA described previously [146]. Briefly, ELISA wells were coated with 100 ul of human MAb 91-5 (anti-HIV p24 antibody) [147] at 0.5 mg/ml at 4°C overnight. The wells were washed 4 times with PBS- 0.05% Tween 20 (PBST) and blocked with 0.2 ml of 5% bovine serum albumin (BSA) in PBST (BSA-PBST) for 6 h at 37°C. After washing the wells again 4 times, 100 ul/well of each supernatant was added. To generate a standard curve for quantitation of p24 in the test samples, recombinant p24 from HIV-1_SF2_ expressed in yeast (Chiron Corp., Emeryville, CA) was reconstituted to contain 250 pg/ml; 100 ul/well of serial two-fold dilutions (250 to 15.6 pg/ml) of each concentration of p24 was added in duplicate wells in the ELISA plate. The plates were incubated at 4°C overnight and then washed 4 times with PBST. One hundred ul of biotinylated human anti-p24 MAb 241-D (which recognizes a different p24 epitope from that recognized by MAb 91-5) [147], [148] diluted 1∶10,000 in PBST-1%BSA was added to each well and ELISA plate incubated at 37°C for 2.5 hr. After subsequent washing, the wells were incubated with streptavidin alkaline phosphatase 1∶1000 (Invitrogen, Carlsbad, CA) for 1 hr at 37°C. Color was developed with an ELISA Amplification System (Invitrogen), and plates were read at 490 nm. The amount of p24 was determined by extrapolating the optical density value of the test sample supernatant on the curve plotted from the values obtained with the recombinant p24. Every experiment was repeated at least twice.

## Supporting Information

Figure S1 ***M. tb*** **genes differentially expressed in blood from HIV- donors only.** Heat maps of (A) upregulated *M. tb* genes and (B) down-regulated *M. tb* genes. Results are shown from *M. tb* grown in blood from 6 HIV- donors (1-6) and 6 HIV+ patients (7-12) with dye flip.
(TIF)

Figure S2 ***M. tb*** **genes differentially expressed in blood from HIV+ patients only.** Heat maps of (A) upregulated *M. tb* genes and (B) down-regulated *M. tb* genes. Results are shown from *M. tb* grown in blood from 6 HIV- donors (1-6) and 6 HIV+ patients (7-12) with dye flip.
(TIF)

Figure S3 ***M. tb*** **genes differentially expressed in blood from both HIV- and HIV+ subjects.** Heat maps of (A) upregulated *M. tb* genes and (B) down-regulated *M. tb* genes. Results are shown from *M. tb* grown in blood from 6 HIV- donors (1-6) and 6 HIV+ patients (7-12) with dye flip.
(TIF)

Figure S4 **Unsupervised Hierarchical Clustergrams of** ***M. tb*** **genes differentially expressed in blood.** Clustergrams of (A) upregulated *M. tb* genes and (B) down-regulated *M. tb* genes from *M. tb* grown in blood from 6 HIV- donors (1-6) and 6 HIV+ patients (7-12) with dye flip. Distance threshold of hierarchical tree was set to 0.75 producing 10 clusters among upregulated genes and 6 clusters among down-regulated genes. Key insert defines numbers in parenthesis (Black- total # genes in cluster; Blue- # of cluster genes affected in HIV- donor blood only, Pink- # of cluster genes affected in HIV+ patient blood only, Orange- # of cluster genes affected in blood from both HIV- and HIV+ donors.
(TIF)

Figure S5 **Differential expression of DevR (DosR) regulon genes in blood and macrophages.** Venn diagrams of numbers of DevR (DosR) regulon genes (A) down-regulated in *M. tb* replicating in blood from HIV- and/or HIV+ subjects with commonly down-regulated genes in the overlap (this study) and (B) upregulated in *M. tb* isolated from THP-1 and/or activated mBMM macrophages with commonly upregulated genes in the overlap (Fontan P et al., 2008; Schnappinger D et al., 2003) [110], [37]. Note: No DevR (DosR) regulon genes were upregulated in either blood environment nor were any down-regulated in either macrophage environment.
(TIF)

Figure S6 **Validation of RNA Amplification Method.** (A) Images of representative *M. tb* chips scanned after hybridization with a mixture of Cy3 and Cy5 labeled cDNA probe derived from unamplified and amplified RNA (aRNA) from *M. tb* grown in HIV- blood and 7H9 broth (reference RNA). (B) Comparison of differentially expressed (≥2-fold) *M. tb* gene profiles in blood with reference to 7H9 broth obtained with unamplified RNA and aRNA; 257/617 (42%) genes identified to be differentially expressed by unamplified RNA were also identified by aRNA. (C) Heat map comparing highly differentially expressed (≥4-fold) *M. tb* gene profiles in blood with reference to 7H9 broth obtained with unamplified and aRNA (three technical replicates including one dye flip); 58/82 (71%) differentially expressed genes identified by unamplified RNA were also identified by aRNA.
(TIF)

Table S1 **Clinical details of HIV+ blood donors and corresponding experiments.**
(DOCX)

Table S2 ***M. tb*** **genes differentially expressed in blood from HIV- donors.**
(PDF)

Table S3 ***M. tb*** **genes differentially expressed in blood from HIV+ patients.**
(PDF)

Table S4 ***M. tb*** **ESX loci-related genes and genes encoding ESAT-6-like proteins outside ESX loci upregulated in blood from HIV- and/or HIV+ subjects.**
(DOCX)

Table S5 **Distribution of differentially expressed DevR (DosR)-regulon genes in blood from HIV- and/or HIV+ subjects in this study and in two published intra-macrophage microarray studies.**
(DOCX)

Table S6 **Primers used for qRT-PCR.**
(DOCX)
