# Supplementary figures and images for: Transcriptional Profiling of Mycobacterium tuberculosis Replicating Ex vivo in Blood from HIV- and HIV+ Subjects
Source: PLoS One. 2014 Apr 22;9(4):e94939. doi: 10.1371/journal.pone.0094939 (PMC3995690; doi:10.1371/journal.pone.0094939)

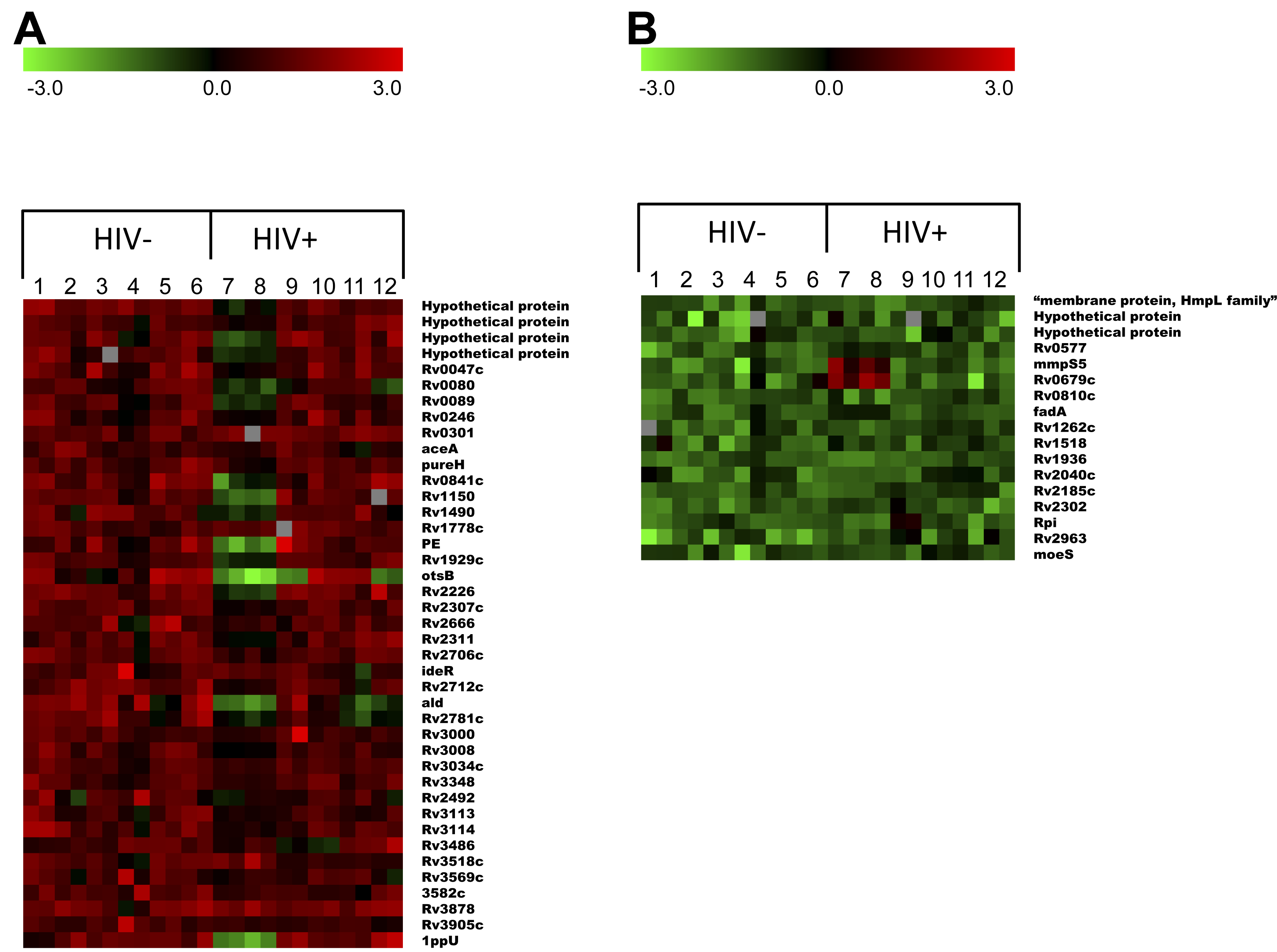

Supplement: Figure S1 — M. tb genes differentially expressed in blood from HIV- donors only. Heat maps of (A) upregulated M. tb genes and (B) down-regulated M. tb genes. Results are shown from M. tb grown in blood from 6 HIV- donors (1-6) and 6 HIV+ patients (7-12) with dye flip. (TIF) [file pone.0094939.s001.tif]

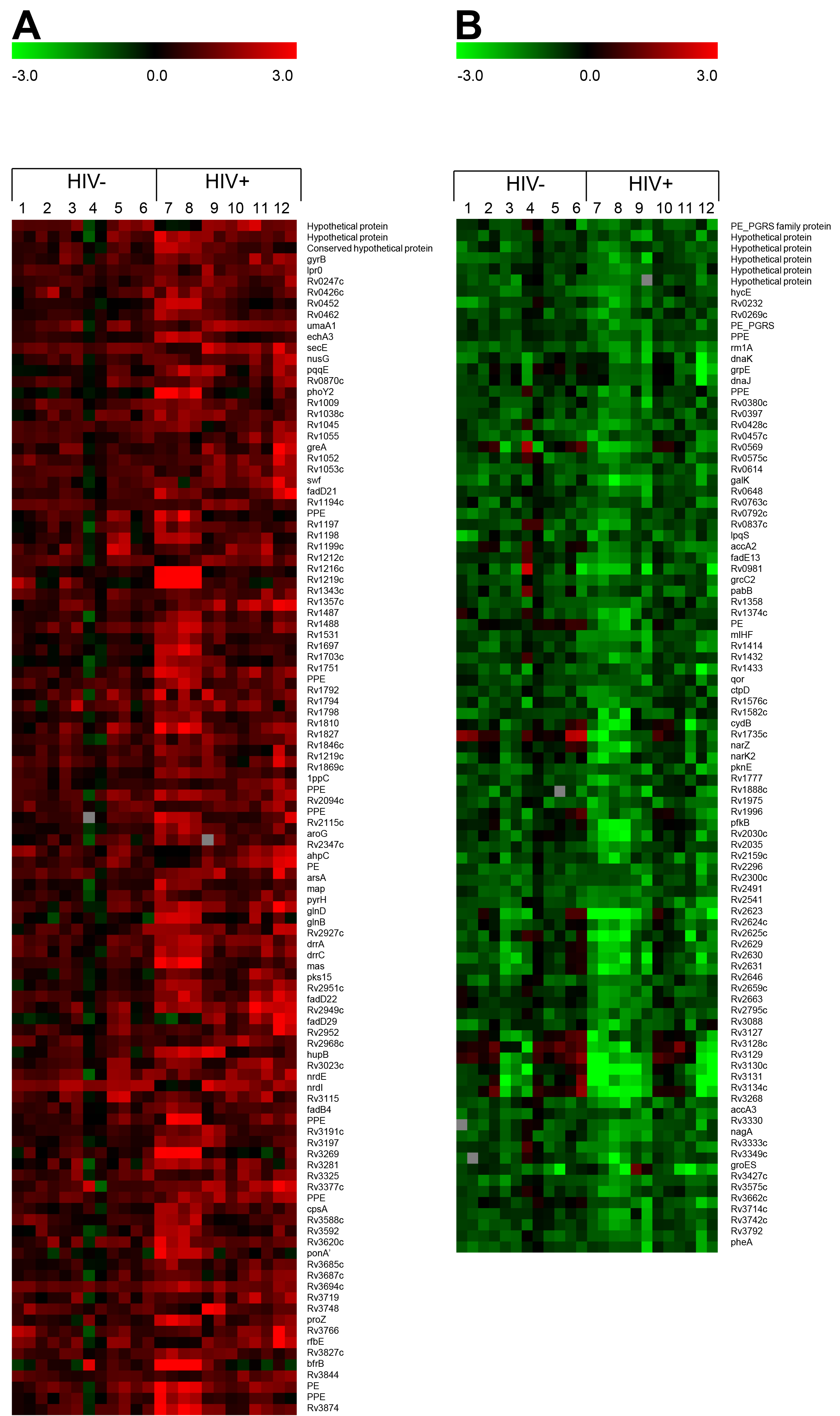

Supplement: Figure S2 — M. tb genes differentially expressed in blood from HIV+ patients only. Heat maps of (A) upregulated M. tb genes and (B) down-regulated M. tb genes. Results are shown from M. tb grown in blood from 6 HIV- donors (1-6) and 6 HIV+ patients (7-12) with dye flip. (TIF) [file pone.0094939.s002.tif]

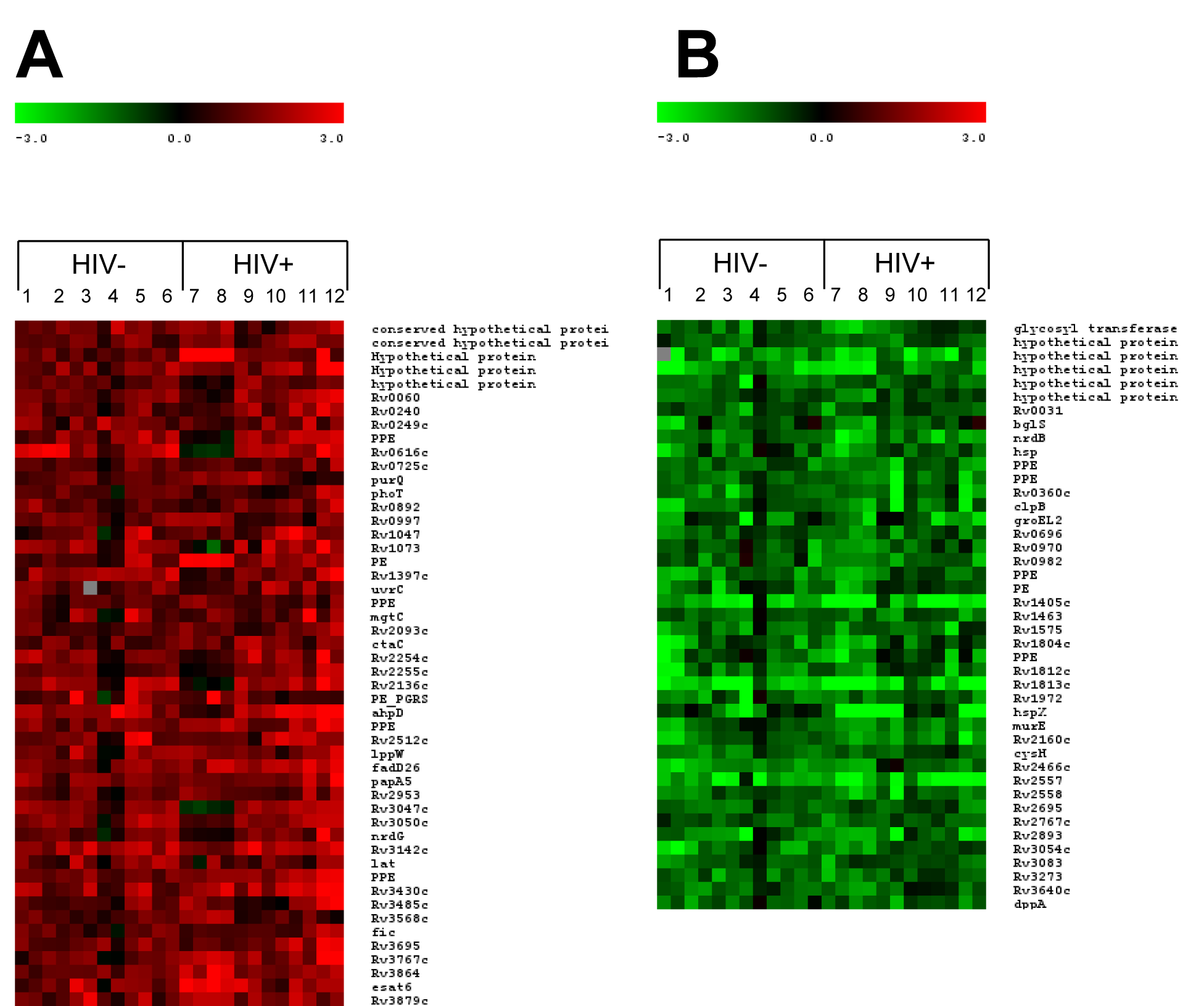

Supplement: Figure S3 — M. tb genes differentially expressed in blood from both HIV- and HIV+ subjects. Heat maps of (A) upregulated M. tb genes and (B) down-regulated M. tb genes. Results are shown from M. tb grown in blood from 6 HIV- donors (1-6) and 6 HIV+ patients (7-12) with dye flip. (TIF) [file pone.0094939.s003.tif]

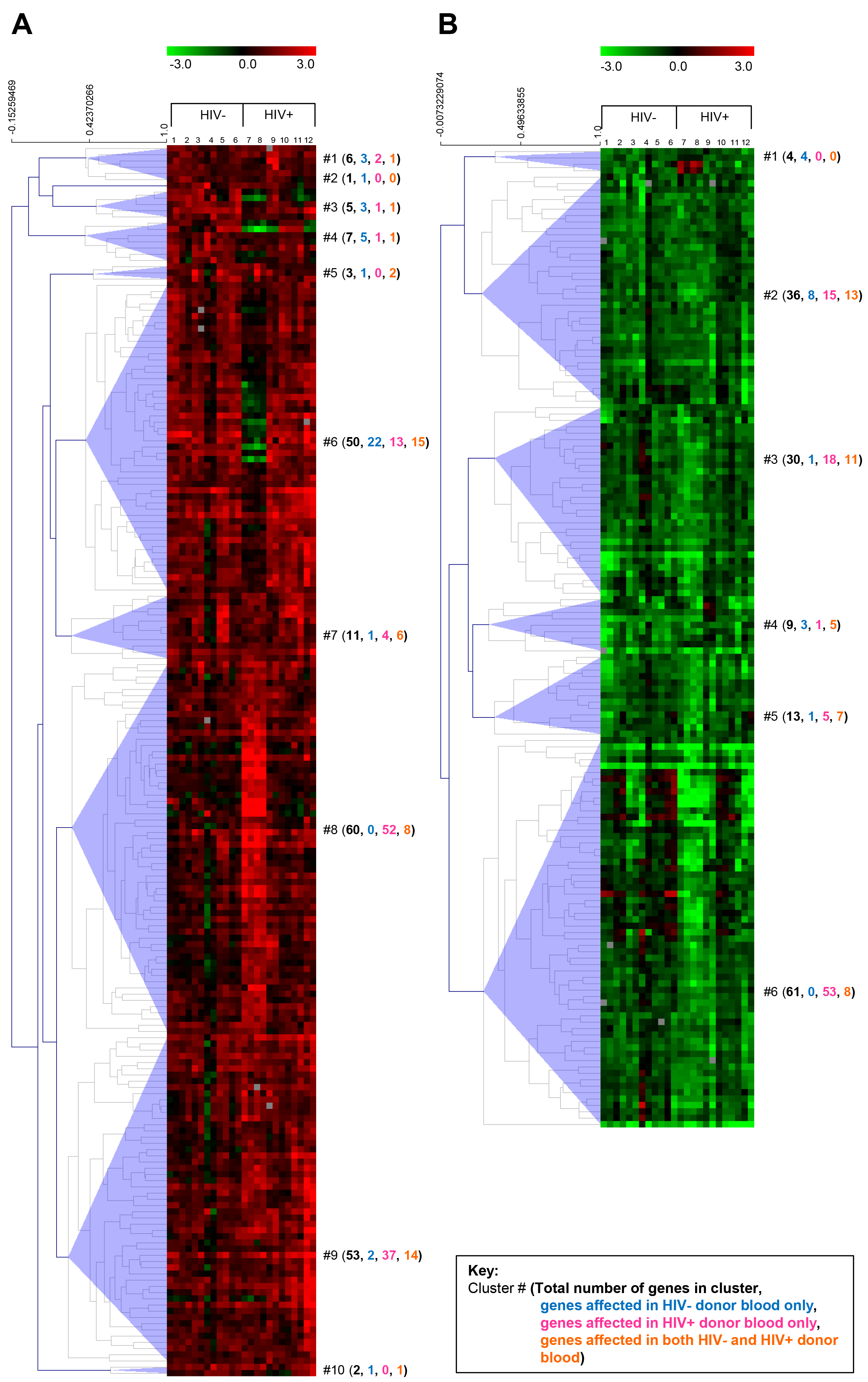

Supplement: Figure S4 — Unsupervised Hierarchical Clustergrams of M. tb genes differentially expressed in blood. Clustergrams of (A) upregulated M. tb genes and (B) down-regulated M. tb genes from M. tb grown in blood from 6 HIV- donors (1-6) and 6 HIV+ patients (7-12) with dye flip. Distance threshold of hierarchical tree was set to 0.75 producing 10 clusters among upregulated genes and 6 clusters among down-regulated genes. Key insert defines numbers in parenthesis (Black- total # genes in cluster; Blue- # of cluster genes affected in HIV- donor blood only, Pink- # of cluster genes affected in HIV+ patient blood only, Orange- # of cluster genes affected in blood from both HIV- and HIV+ donors. (TIF) [file pone.0094939.s004.tif]

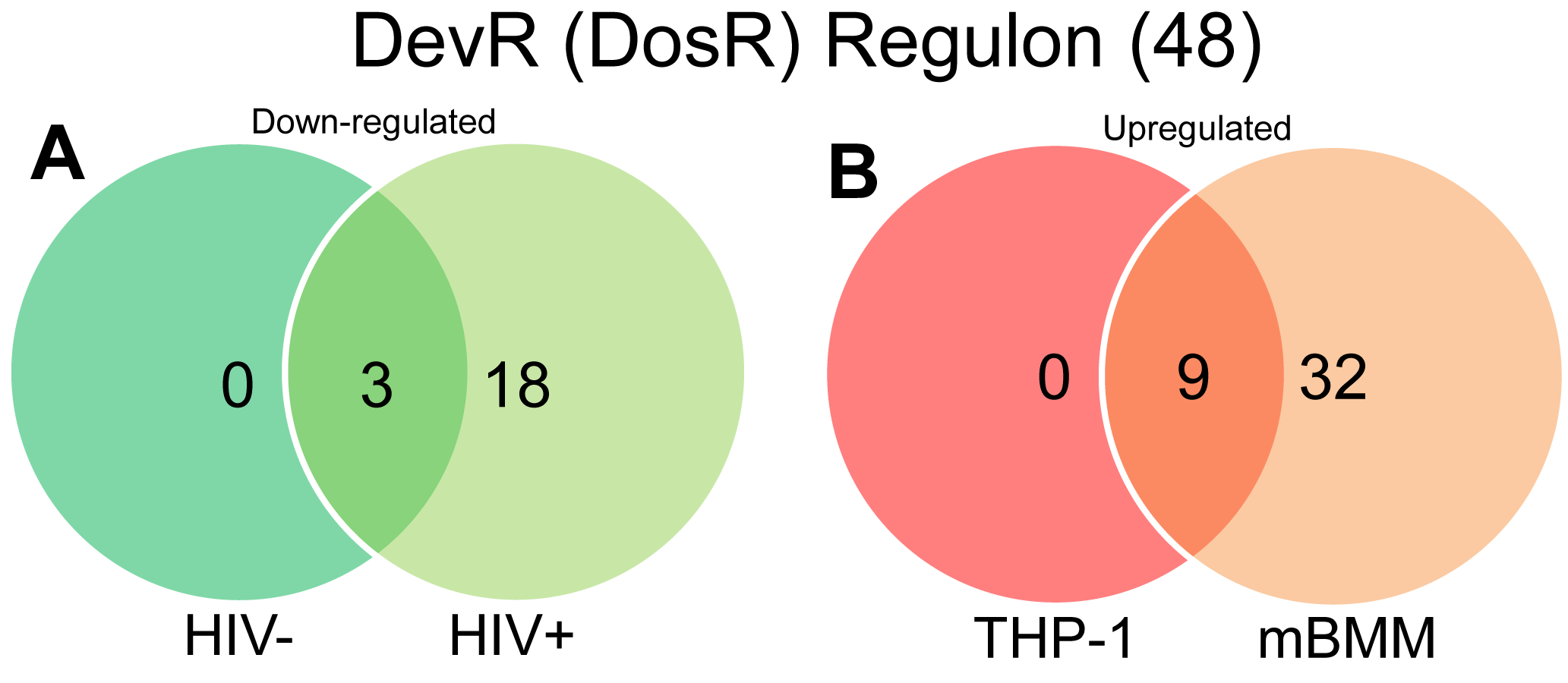

Supplement: Figure S5 — Differential expression of DevR (DosR) regulon genes in blood and macrophages. Venn diagrams of numbers of DevR (DosR) regulon genes (A) down-regulated in M. tb replicating in blood from HIV- and/or HIV+ subjects with commonly down-regulated genes in the overlap (this study) and (B) upregulated in M. tb isolated from THP-1 and/or activated mBMM macrophages with commonly upregulated genes in the overlap (Fontan P et al., 2008; Schnappinger D et al., 2003) [110], [37]. Note: No DevR (DosR) regulon genes were upregulated in either blood environment nor were any down-regulated in either macrophage environment. (TIF) [file pone.0094939.s005.tif]

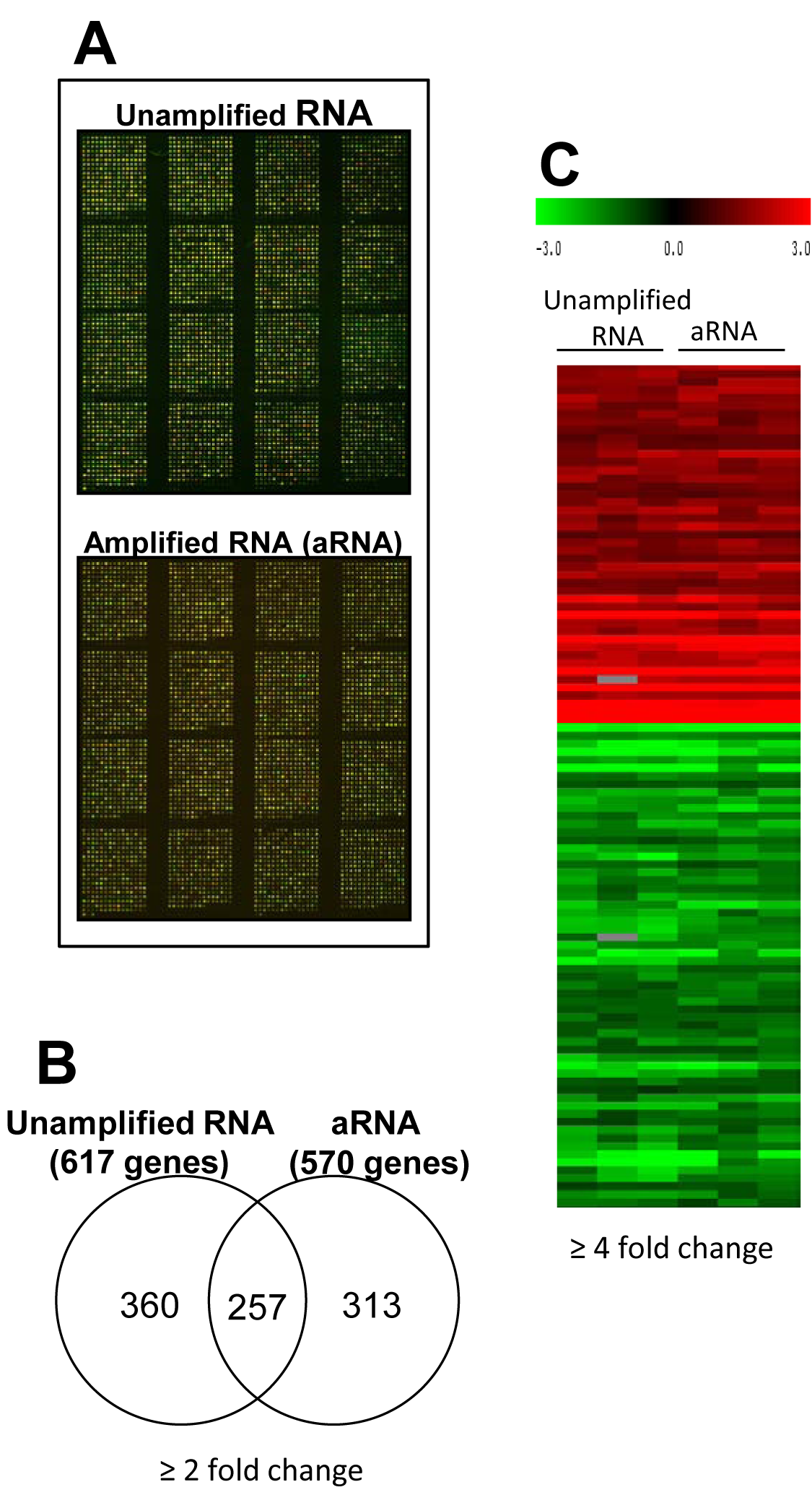

Supplement: Figure S6 — Validation of RNA Amplification Method. (A) Images of representative M. tb chips scanned after hybridization with a mixture of Cy3 and Cy5 labeled cDNA probe derived from unamplified and amplified RNA (aRNA) from M. tb grown in HIV- blood and 7H9 broth (reference RNA). (B) Comparison of differentially expressed (≥2-fold) M. tb gene profiles in blood with reference to 7H9 broth obtained with unamplified RNA and aRNA; 257/617 (42%) genes identified to be differentially expressed by unamplified RNA were also identified by aRNA. (C) Heat map comparing highly differentially expressed (≥4-fold) M. tb gene profiles in blood with reference to 7H9 broth obtained with unamplified and aRNA (three technical replicates including one dye flip); 58/82 (71%) differentially expressed genes identified by unamplified RNA were also identified by aRNA. (TIF) [file pone.0094939.s006.tif]
