# Supplementary material for: Transcriptional Profiling of Mycobacterium tuberculosis Replicating Ex vivo in Blood from HIV- and HIV+ Subjects
Source: PLoS One. 2014 Apr 22;9(4):e94939. doi: 10.1371/journal.pone.0094939 (PMC3995690; doi:10.1371/journal.pone.0094939)
Supplement: Table S1 — Clinical details of HIV+ blood donors and corresponding experiments. (DOCX) [file pone.0094939.s007.docx]

**Table S1.** Clinical details of HIV+ blood donors and corresponding experiments

| **Patient #** | **CD4+ T cell count (cells/mm^3^)** | **Viral Load (copies/ml)** | **CFU** | **Microarray** | **qRT-PCR** |
| --- | --- | --- | --- | --- | --- |
| H11 | 246 | <75 | **X** |  |  |
| H12 | 282 | <75 | **X** |  |  |
| H16 | 903 | <75 |  | **X** |  |
| H20 | 348 | 16177 (high) |  | **X** |  |
| H24 | 79 | <75 | **X** | **X** | **X** |
| H25 | 655 | <75 | **X** |  |  |
| H26 | 325 | <75 | **X** |  |  |
| H27 | 183 | 40117 (high) | **X** |  |  |
| H28 | 856 | <75 | **X** | **X** | **X** |
| H29 | 400 | <75 | **X** |  |  |
| H30 | 806 | <75 | **X** | **X** | **X** |
| H33 | 567 | <75 | **X** |  |  |
| H34 | 660 | 185 (low) | **X** | **X** | **X** |
| H35 | 696 | <75 | **X** |  | **X** |
| H36 | 282 | <75 | **X** |  | **X** |
| H37 | 376 | <75 | **X** |  |  |
| H38 | 269 | <75 | **X** |  |  |
