# Supplementary material for: Transcriptional Profiling of Mycobacterium tuberculosis Replicating Ex vivo in Blood from HIV- and HIV+ Subjects
Source: PLoS One. 2014 Apr 22;9(4):e94939. doi: 10.1371/journal.pone.0094939 (PMC3995690; doi:10.1371/journal.pone.0094939)
Supplement: Table S2 — M. tb genes differentially expressed in blood from HIV- donors. (PDF) [file pone.0094939.s008.pdf]

Table S2. *M. tb* genes differentially expressed in HIV- blood

Upregulated in HIV- blood			Down-regulated in HIV- blood		
Name	ID	Ave fold change	Name	ID	Ave fold change
Rv0616c	Rv0616c	4.255205	Rv1813c	Rv1813c	-5.93924
Rv3142c	Rv3142c	3.798676	Rv1405c	Rv1405c	-4.45885
ahpD	Rv2429	3.776029	Rv2557	Rv2557	-4.35012
Rv1397c	Rv1397c	3.507397	hypothetic	ORF04548	-4.3037
Rv2136c	Rv2136c	3.272688	hypothetic	ORF03834	-3.58952
Rv1073	Rv1073	3.056659	Rv2466c	Rv2466c	-3.55825
Rv3050c	Rv3050c	2.886824	Rv1812c	Rv1812c	-3.00419
hypothetic	ORF02013	2.848007	Rv3054c	Rv3054c	-2.97855
Rv3047c	Rv3047c	2.845427	PPE	Rv1168c	-2.89417
PPE	Rv0442c	2.800798	Rv2963	Rv2963	-2.79852
Rv3430c	Rv3430c	2.740101	Rv2893	Rv2893	-2.7273
papA5	Rv2939	2.738938	Rv1972	Rv1972	-2.67192
ald	Rv2780	2.717154	Rv1804c	Rv1804c	-2.65388
Rv2254c	Rv2254c	2.690696	hypothetic	ORF05389	-2.6301
Rv2712c	Rv2712c	2.647327	Rv1575	Rv1575	-2.60292
Rv0249c	Rv0249c	2.642459	cysH	Rv2392	-2.53469
Rv2226	Rv2226	2.609259	hypothetic	ORFD0123	-2.51504
fadD26	Rv2930	2.598588	Rv0360c	Rv0360c	-2.49388
Hypothetic	ORF08412	2.577407	clpB	Rv0384c	-2.41739
conserved	ORF01755	2.573879	Rv2160c	Rv2160c	-2.40777
PPE	Rv2430c	2.564311	nrdB	Rv0233	-2.38213
lat	Rv3290c	2.561843	Rv3273	Rv3273	-2.31768
Rv2512c	Rv2512c	2.55995	mmpS5	Rv0677c	-2.31387
Rv0060	Rv0060	2.556688	groEL2	Rv0440	-2.31305
Rv3878	Rv3878	2.554688	PPE	Rv1807	-2.30627
Rv0841c	Rv0841c	2.553322	Rv2695	Rv2695	-2.29248
esat6	Rv3875	2.55067	Rv0577	Rv0577	-2.25209
lppW	Rv2905	2.514516	Rv2302	Rv2302	-2.23917
Rv3879c	Rv3879c	2.484001	Rv3640c	Rv3640c	-2.22357
Rv0047c	Rv0047c	2.45954	PPE	Rv0280	-2.21733
Rv3114	Rv3114	2.409916	PPE	Rv0304c	-2.21425
Rv3864	Rv3864	2.392678	PE	Rv1169c	-2.2108
Rv2781c	Rv2781c	2.383829	Rv2558	Rv2558	-2.20379
PPE	Rv3429	2.382233	glycosyl tra	ORF00811	-2.15955
Rv0997	Rv0997	2.376553	hsp	Rv0251c	-2.15134
ideR	Rv2711	2.364263	hspX	Rv2031c	-2.15042
uvrC	Rv1420	2.354721	Rv3083	Rv3083	-2.14988
otsB	Rv2006	2.334549	Rv0810c	Rv0810c	-2.09497
PE_PGRS	Rv2396	2.32801	murE	Rv2158c	-2.09163
Rv3695	Rv3695	2.327796	Rv0696	Rv0696	-2.08619
Rv2706c	Rv2706c	2.327095	bglS	Rv0186	-2.06376
Rv2307c	Rv2307c	2.319043	Rv0982	Rv0982	-2.06177
lppU	Rv2784c	2.303224	dppA	Rv3666c	-2.04321
Rv3582c	Rv3582c	2.241111	Rv2185c	Rv2185c	-2.03877

hypothetic	ORF04431	2.186514
conserved	ORF01756	2.182176
hypothetic	ORFD0084	2.177318
hypothetic	ORFD0392	2.177141
Rv1150	Rv1150	2.17714
purM	Rv0809	2.162973
mgtC	Rv1811	2.158824
Rv0240	Rv0240	2.150602
Rv2666	Rv2666	2.149298
Hypotheti	ORF08410	2.141418
Rv3008	Rv3008	2.13738
PE	Rv1195	2.132019
Rv0301	Rv0301	2.126702
Rv2311	Rv2311	2.125549
Rv1490	Rv1490	2.121841
purQ	Rv0788	2.116452
PE	Rv1806	2.111235
Rv0892	Rv0892	2.074808
nrdG	Rv3048c	2.064686
Rv3518c	Rv3518c	2.062638
Rv0725c	Rv0725c	2.059624
PPE	Rv1787	2.042
Rv1047	Rv1047	2.039708
Rv3767c	Rv3767c	2.037948
Rv3905c	Rv3905c	2.035978
Rv2255c	Rv2255c	2.03412
Rv1778c	Rv1778c	2.033915
Rv3348	Rv3348	2.028576
Rv0089	Rv0089	2.027071
Rv0080	Rv0080	2.026558
Rv3569c	Rv3569c	2.021558
Rv1929c	Rv1929c	2.017994
Rv0246	Rv0246	2.00984
Rv3485c	Rv3485c	2.006529
ctaC	Rv2200c	2.006136
Rv3034c	Rv3034c	2.003161
phoT	Rv0820	1.997833
Rv3000	Rv3000	1.996734
Rv3113	Rv3113	1.993811
Rv2492	Rv2492	1.989315
Rv2953	Rv2953	1.987983
fic	Rv3641c	1.986779
Rv3568c	Rv3568c	1.979714
Rv2093c	Rv2093c	1.971662
hypothetic	ORF02708	1.953795
aceA	Rv0467	1.952831
Rv3486	Rv3486	1.951248

Rv1936	Rv1936	-2.03746
Rv0031	Rv0031	-2.01749
Rv1463	Rv1463	-2.01013
hypothetic	ORF01236	-2.0036
Rv1518	Rv1518	-1.99489
hypothetic	ORFD0249	-1.98822
rpi	Rv2465c	-1.9823
fadA	Rv0859	-1.98082
hypothetic	ORFD0167	-1.97842
membrane	ORF00814	-1.97306
Rv2767c	Rv2767c	-1.97299
Rv0679c	Rv0679c	-1.96717
Rv1262c	Rv1262c	-1.96584
Rv0970	Rv0970	-1.96183
moeZ	Rv3206c	-1.95942
Rv2040c	Rv2040c	-1.95316
