# Supplementary material for: Transcriptional Profiling of Mycobacterium tuberculosis Replicating Ex vivo in Blood from HIV- and HIV+ Subjects
Source: PLoS One. 2014 Apr 22;9(4):e94939. doi: 10.1371/journal.pone.0094939 (PMC3995690; doi:10.1371/journal.pone.0094939)
Supplement: Table S3 — M. tb genes differentially expressed in blood from HIV+ patients. (PDF) [file pone.0094939.s009.pdf]

Table S3. *M. tb* genes differentially expressed in HIV+ blood

Upregulated in HIV+ blood			Down-regulated in HIV+ blood		
Name	ID	Ave fold change	Name	ID	Ave fold change
esat6	Rv3875	4.976237	Rv1813c	Rv1813c	-9.67657
Rv3430c	Rv3430c	4.959644	Rv1405c	Rv1405c	-8.36637
PE	Rv1195	4.489464	Rv2557	Rv2557	-6.47946
fadD26	Rv2930	4.389924	hspX	Rv2031c	-6.34663
Hypotheti	ORF08410	4.345157	Rv3130c	Rv3130c	-5.56399
Rv2949c	Rv2949c	4.172022	Rv3131	Rv3131	-5.19323
ahpD	Rv2429	4.101131	Rv2623	Rv2623	-4.72768
Rv3767c	Rv3767c	4.0588	hypothetic	ORF03834	-4.64812
Rv3142c	Rv3142c	3.817115	hypothetic	ORF04548	-3.81268
Rv3864	Rv3864	3.801844	nrdB	Rv0233	-3.68611
PPE	Rv3429	3.748489	Rv3129	Rv3129	-3.46173
Rv1810	Rv1810	3.732326	Rv3134c	Rv3134c	-3.39822
PPE	Rv2430c	3.730391	Rv2893	Rv2893	-3.37232
Hypotheti	ORF08412	3.716524	Rv2160c	Rv2160c	-3.29929
glnD	Rv2918c	3.512662	Rv2625c	Rv2625c	-3.15547
PE	Rv3872	3.427545	Rv0981	Rv0981	-3.12252
Rv3874	Rv3874	3.384901	PPE	Rv0304c	-3.06152
Rv3879c	Rv3879c	3.361989	murE	Rv2158c	-3.03047
Rv3377c	Rv3377c	3.25285	Rv2631	Rv2631	-3.02703
Rv1751	Rv1751	3.190597	Rv2630	Rv2630	-3.01026
Rv1357c	Rv1357c	3.138493	Rv2030c	Rv2030c	-3.00915
Rv1219c	Rv1219c	3.005053	galK	Rv0620	-2.97531
Rv2512c	Rv2512c	2.983203	clpB	Rv0384c	-2.96175
papA5	Rv2939	2.971018	Rv2558	Rv2558	-2.9496
fadD22	Rv2948c	2.956922	Rv0360c	Rv0360c	-2.86971
drrA	Rv2936	2.955693	PPE	Rv0280	-2.73405
Rv1047	Rv1047	2.951562	groEL2	Rv0440	-2.69054
PPE	Rv3425	2.948718	Rv2541	Rv2541	-2.68097
Rv3695	Rv3695	2.922076	rmlA	Rv0334	-2.64595
Rv3694c	Rv3694c	2.907098	Rv0982	Rv0982	-2.64595
Rv2094c	Rv2094c	2.891362	Rv3268	Rv3268	-2.61764
Rv3050c	Rv3050c	2.875673	mIHF	Rv1388	-2.57073
lppW	Rv2905	2.864516	Rv2629	Rv2629	-2.56167
Rv0060	Rv0060	2.803709	PPE	Rv1168c	-2.50788
hypothetic	ORF04372	2.802435	Rv2159c	Rv2159c	-2.47203
Rv0240	Rv0240	2.788122	hsp	Rv0251c	-2.47155
conserved	ORF01755	2.763499	nagA	Rv3332	-2.44959
secE	Rv0638	2.7512	Rv0970	Rv0970	-2.44775
drrC	Rv2938	2.742778	narX	Rv1736c	-2.4425
hupB	Rv2986c	2.734216	Rv3330	Rv3330	-2.44193
ctaC	Rv2200c	2.727267	PPE	Rv1807	-2.43664
Rv1488	Rv1488	2.720143	glycosyl tr	ORF00811	-2.43001
mas	Rv2940c	2.718753	groES	Rv3418c	-2.42987
Rv2254c	Rv2254c	2.713337	Rv1804c	Rv1804c	-2.41208

conserved	ORF01756	2.706386	Rv3662c	Rv3662c	-2.39067
proX	Rv3759c	2.700999	pfkB	Rv2029c	-2.38388
PPE	Rv0442c	2.698388	Rv2624c	Rv2624c	-2.38275
pqqE	Rv0693	2.661396	Rv2466c	Rv2466c	-2.38097
Rv3485c	Rv3485c	2.630343	Rv0696	Rv0696	-2.35659
Rv3620c	Rv3620c	2.627931	dnaK	Rv0350	-2.35506
PPE	Rv3159c	2.613973	Rv1463	Rv1463	-2.35488
Rv1397c	Rv1397c	2.613449	Rv3128c	Rv3128c	-2.34861
arsA	Rv2684	2.610015	Rv1414	Rv1414	-2.34362
umaA1	Rv0469	2.602709	Rv1575	Rv1575	-2.34145
Rv2136c	Rv2136c	2.600616	Rv3333c	Rv3333c	-2.34021
Rv2927c	Rv2927c	2.575851	Rv3427c	Rv3427c	-2.33202
Rv3281	Rv3281	2.560285	Rv1975	Rv1975	-2.31761
nrdE	Rv3051c	2.550883	narK2	Rv1737c	-2.31469
Rv1216c	Rv1216c	2.5287	Rv0428c	Rv0428c	-2.31379
bfrB	Rv3841	2.521432	Rv1433	Rv1433	-2.30848
fadD21	Rv1185c	2.516755	cydB	Rv1622c	-2.29774
Rv2951c	Rv2951c	2.514535	Rv1972	Rv1972	-2.29596
zwf	Rv1121	2.510375	hypothetic	ORF06004	-2.28919
phoY2	Rv0821c	2.508742	Rv3349c	Rv3349c	-2.28747
gyrB	Rv0005	2.499588	pknE	Rv1743	-2.28703
Rv0616c	Rv0616c	2.474094	fadE13	Rv0975c	-2.28607
Rv2115c	Rv2115c	2.451312	accA2	Rv0973c	-2.28477
PE	Rv2431c	2.433467	PE_PGRS	Rv0278c	-2.2765
Rv3766	Rv3766	2.428588	Rv1812c	Rv1812c	-2.27106
Rv3023c	Rv3023c	2.405135	Rv3792	Rv3792	-2.26363
mgtC	Rv1811	2.404265	Rv0569	Rv0569	-2.25762
Rv1792	Rv1792	2.399341	Rv2659c	Rv2659c	-2.24817
Rv1794	Rv1794	2.396671	dppA	Rv3666c	-2.24464
Rv1052	Rv1052	2.39078	Rv2767c	Rv2767c	-2.23601
Rv2953	Rv2953	2.387233	Rv3273	Rv3273	-2.23151
Rv1038c	Rv1038c	2.385125	Rv3714c	Rv3714c	-2.22536
Rv0997	Rv0997	2.371195	grpE	Rv0351	-2.22378
nrdI	Rv3052c	2.36912	Rv2795c	Rv2795c	-2.21878
Rv2093c	Rv2093c	2.368468	Rv0837c	Rv0837c	-2.20595
purQ	Rv0788	2.359463	Rv3742c	Rv3742c	-2.20547
uvrC	Rv1420	2.35176	Rv0232	Rv0232	-2.20069
Rv1073	Rv1073	2.351382	Rv1374c	Rv1374c	-2.19773
Rv1697	Rv1697	2.321978	Rv3088	Rv3088	-2.19709
aroG	Rv2178c	2.305938	Rv3054c	Rv3054c	-2.19277
Rv0892	Rv0892	2.303099	Rv0614	Rv0614	-2.18908
Rv2255c	Rv2255c	2.302579	Rv1582c	Rv1582c	-2.18636
Rv0249c	Rv0249c	2.294157	Rv0380c	Rv0380c	-2.17982
rfbE	Rv3782	2.284707	hypothetic	ORF05389	-2.15679
PPE	Rv1790	2.269015	Rv1888c	Rv1888c	-2.15645
Rv0247c	Rv0247c	2.265548	hypothetic	ORFD0108	-2.15492
cpsA	Rv3484	2.263381	hycE	Rv0087	-2.14671

nusG	Rv0639	2.259796
conserved	ORF05520	2.253155
Rv1198	Rv1198	2.252633
Rv3197	Rv3197	2.250401
Rv3269	Rv3269	2.241688
Rv3568c	Rv3568c	2.229786
nrdG	Rv3048c	2.222497
Rv3827c	Rv3827c	2.21794
fic	Rv3641c	2.217263
Rv3047c	Rv3047c	2.213769
Rv1053c	Rv1053c	2.207774
Rv3719	Rv3719	2.205862
greA	Rv1080c	2.202103
Rv0462	Rv0462	2.19704
lppC	Rv1911c	2.16871
Rv0725c	Rv0725c	2.159039
glnB	Rv2919c	2.157741
Rv0426c	Rv0426c	2.150136
Rv2952	Rv2952	2.147673
PE_PGRS	Rv2396	2.142506
PPE	Rv1787	2.140373
Rv1487	Rv1487	2.133779
Rv0452	Rv0452	2.133047
Rv1869c	Rv1869c	2.130302
Rv1827	Rv1827	2.126554
lprO	Rv0179c	2.125097
Rv3191c	Rv3191c	2.121319
ponA'	Rv3682	2.113719
hypothetic	ORF03618	2.10909
echA3	Rv0632c	2.107783
PPE	Rv3873	2.106112
Rv3748	Rv3748	2.099974
Rv3115	Rv3115	2.098414
Rv2347c	Rv2347c	2.071
lat	Rv3290c	2.062367
Rv1197	Rv1197	2.061444
fadD29	Rv2950c	2.06061
Rv1045	Rv1045	2.058474
Rv1343c	Rv1343c	2.054565
Rv2968c	Rv2968c	2.053601
Rv3592	Rv3592	2.039422
Rv3588c	Rv3588c	2.037643
Rv1212c	Rv1212c	2.036569
Rv2091c	Rv2091c	2.031291
Rv3687c	Rv3687c	2.030228
Rv1199c	Rv1199c	2.029963
Rv1009	Rv1009	2.027337

hypothetic	ORF01236	-2.14476
PE_PGRS f	ORF03389	-2.14243
Rv2491	Rv2491	-2.13377
bglS	Rv0186	-2.12663
hypothetic	ORF05172	-2.10869
Rv1735c	Rv1735c	-2.10702
hypothetic	ORF00230	-2.10611
Rv1432	Rv1432	-2.10589
lpqS	Rv0847	-2.10476
Rv1996	Rv1996	-2.10463
Rv0457c	Rv0457c	-2.0873
Rv0575c	Rv0575c	-2.08486
dnaJ	Rv0352	-2.0834
hypothetic	ORFD0167	-2.06956
Rv2296	Rv2296	-2.06918
Rv0648	Rv0648	-2.06752
hypothetic	ORFD0057	-2.06523
PE	Rv1386	-2.05629
Rv3640c	Rv3640c	-2.05205
Rv1777	Rv1777	-2.04882
Rv0397	Rv0397	-2.04723
ctpD	Rv1469	-2.04687
accA3	Rv3285	-2.03901
Rv3083	Rv3083	-2.0356
Rv2695	Rv2695	-2.03547
Rv2035	Rv2035	-2.03218
pheA	Rv3838c	-2.03128
Rv1576c	Rv1576c	-2.0243
Rv3127	Rv3127	-2.02287
Rv2300c	Rv2300c	-2.01381
PE	Rv1169c	-2.00925
pabB	Rv1005c	-2.00533
Rv0269c	Rv0269c	-1.99905
Rv0031	Rv0031	-1.99689
Rv2646	Rv2646	-1.99287
Rv1358	Rv1358	-1.99228
Rv2663	Rv2663	-1.99145
PPE	Rv0305c	-1.98054
Rv0763c	Rv0763c	-1.979
Rv3575c	Rv3575c	-1.97607
Rv0792c	Rv0792c	-1.9746
cysH	Rv2392	-1.96864
grcC2	Rv0989c	-1.96461
PPE	Rv0355c	-1.96171
qor	Rv1454c	-1.96048

pks15	Rv2947c	2.023439
phoT	Rv0820	2.019955
ahpC	Rv2428	2.018173
fadB4	Rv3141	2.01351
Rv1798	Rv1798	2.005583
hypothetic	ORFD0392	2.004435
Rv1194c	Rv1194c	1.996417
Rv1703c	Rv1703c	1.995277
Rv3325	Rv3325	1.982739
Rv0870c	Rv0870c	1.978637
Rv3685c	Rv3685c	1.978247
pyrH	Rv2883c	1.971007
Rv3844	Rv3844	1.96681
Rv1531	Rv1531	1.966667
PPE	Rv1196	1.964563
PPE	Rv1917c	1.96339
Rv1055	Rv1055	1.963002
map	Rv2861c	1.962614
Rv1846c	Rv1846c	1.962289
PPE	Rv2108	1.960106
