# Supplementary material for: Transcriptional Profiling of Mycobacterium tuberculosis Replicating Ex vivo in Blood from HIV- and HIV+ Subjects
Source: PLoS One. 2014 Apr 22;9(4):e94939. doi: 10.1371/journal.pone.0094939 (PMC3995690; doi:10.1371/journal.pone.0094939)
Supplement: Table S4 — M. tb ESX loci-related genes and genes encoding ESAT-6-like proteins outside ESX loci upregulated in blood from HIV- and/or HIV+ subjects. (DOCX) [file pone.0094939.s010.docx]

**Table S4.** *M. tb* ESX loci-related genes and genes encoding ESAT-6-like proteins outside ESX loci upregulated in blood from HIV- and/or HIV+ subjects.

| **Gene Description** | **ESX-1 locus** | | **ESX-5 locus** | | **Outside of any ESX loci** | |
| --- | --- | --- | --- | --- | --- | --- |
|  | **HIV-** | **HIV+** | **HIV-** | **HIV+** | **HIV-** | **HIV+** |
| **ESAT-6 homologue** | Rv3875 (*esat-6*) | Rv3875 (*esat-6*) | - | - | Rv3905c (*esxF*) | Rv1198 (*esxL*) |
| **CFP-10 homologue** | - | Rv3874 (*cfp-10*) | - | Rv1792 (*esxM*) | - | Rv3620c (*esxW*) |
|  |  |  |  |  |  | Rv1038c (*esxJ*) |
|  |  |  |  |  |  | Rv1197 (*esxK*) |
|  |  |  |  |  |  | Rv2347c (*esxP*) |
| **PE/PPE within locus** | - | Rv3872 (*pe35*) | Rv1787 (*ppe25*) | Rv1787 (*ppe25*) | N/A | N/A |
|  |  | Rv3873 (*ppe68*) |  | Rv1790 (*ppe27*) |  |  |
| **Other locus-specific genes** | Rv3864 (*espE*) | Rv3864 (*espE*) | - | Rv1794 | N/A | N/A |
|  | Rv3879c (*espK*) | Rv3879c (*espK*) |  | Rv1798 (*eccA5*) |  |  |
|  | Rv3878 (*espJ*) |  |  |  |  |  |

Negative sign (-) indicates no gene differentially expressed in this category. N/A indicates not applicable.
