# Supplementary material for: Transcriptional Profiling of Mycobacterium tuberculosis Replicating Ex vivo in Blood from HIV- and HIV+ Subjects
Source: PLoS One. 2014 Apr 22;9(4):e94939. doi: 10.1371/journal.pone.0094939 (PMC3995690; doi:10.1371/journal.pone.0094939)
Supplement: Table S5 — Distribution of differentially expressed DevR (DosR)-regulon genes in blood from HIV- and/or HIV+ subjects in this study and in two published intra-macrophage microarray studies. (DOCX) [file pone.0094939.s011.docx]

**Table S5.** Distribution of differentially expressed DevR (DosR)-regulon genes in blood from HIV- and/or HIV+ subjects in this study and in two published intra-macrophage microarray studies.

| **DevR regulon gene** | **HIV- blood** | **HIV+ blood** | **THP-1** | **mBMM (activated)** |
| --- | --- | --- | --- | --- |
| (All 48 listed) | (This Study) | (This Study) | (Fontan P et al., 2008) | (Schnappinger D et al. 2003) |
| Rv0079 | NDE | NDE | NDE | Up |
| Rv0080 | NDE | NDE | Up | Up |
| Rv0081 | NDE | NDE | Up | Up |
| Rv0569 | NDE | Down | NDE | Up |
| Rv0570 (*nrdZ*) | NDE | NDE | NDE | NDE |
| Rv0571c | NDE | NDE | NDE | Up |
| Rv0572c | NDE | NDE | NDE | Up |
| Rv0573c (*pncB2*) | NDE | NDE | NDE | NDE |
| Rv0574c | NDE | NDE | NDE | Up |
| Rv1733c | NDE | NDE | NDE | Up |
| Rv1734c | NDE | NDE | NDE | Up |
| Rv1735c | NDE | Down | NDE | Up |
| Rv1736c (*narX*) | NDE | Down | NDE | NDE |
| Rv1737c (*narK2*) | NDE | Down | NDE | Up |
| Rv1738 | NDE | NDE | NDE | Up |
| Rv1812c | Down | Down | NDE | NDE |
| Rv1813c | Down | Down | NDE | Up |
| Rv1996 | NDE | Down | NDE | Up |
| Rv1997 (*ctpF*) | NDE | NDE | NDE | Up |
| Rv1998c | NDE | NDE | NDE | NDE |
| Rv2003c | NDE | NDE | NDE | Up |
| Rv2004c | NDE | NDE | NDE | NDE |
| Rv2005c | NDE | NDE | NDE | Up |
| Rv2006 (*ostB1*) | NDE | NDE | NDE | Up |
| Rv2007c | NDE | NDE | NDE | Up |
| Rv2028c | NDE | NDE | Up | Up |
| Rv2029c (*pfkB*) | NDE | NDE | Up | Up |
| Rv2030c | NDE | Down | Up | Up |
| Rv2031c (*hspX*) | Down | Down | Up | Up |
| Rv2032 (*acg*) | NDE | NDE | Up | Up |
| Rv2623 (TB31.7) | NDE | Down | NDE | Up |
| Rv2624c | NDE | Down | NDE | Up |
| Rv2625c | NDE | Down | NDE | Up |
| Rv2626c (*hrp1*) | NDE | NDE | Up | Up |
| Rv2627c | NDE | NDE | NDE | Up |
| Rv2628 | NDE | NDE | NDE | Up |
| Rv2629 | NDE | Down | NDE | Up |
| Rv2630 | NDE | Down | NDE | Up |
| Rv2631 | NDE | Down | NDE | NDE |
| Rv3126c | NDE | NDE | NDE | Up |
| Rv3127 | NDE | Down | NDE | Up |
| Rv3128c | NDE | Down | NDE | Up |
| Rv3129 | NDE | Down | NDE | Up |
| Rv3130c (*tgs1*) | NDE | Down | NDE | Up |
| Rv3131 | NDE | Down | NDE | Up |
| Rv3132c (*devS*) | NDE | NDE | NDE | Up |
| Rv3133c (*devR*) | NDE | NDE | Up | Up |
| Rv3134c | NDE | Down | NDE | Up |

“Up” indicates upregulated, “Down” indicates down-regulated, “NDE” indicates “not differentially expressed”.
