# Supplementary material for: Transcriptional Profiling of Mycobacterium tuberculosis Replicating Ex vivo in Blood from HIV- and HIV+ Subjects
Source: PLoS One. 2014 Apr 22;9(4):e94939. doi: 10.1371/journal.pone.0094939 (PMC3995690; doi:10.1371/journal.pone.0094939)
Supplement: Table S6 — Primers used for qRT-PCR. (DOCX) [file pone.0094939.s012.docx]

**Table S6**. Primers used for qRT-PCR

| **Gene** | **Direction** | **Primer sequence** |
| --- | --- | --- |
| Rv3875 | Forward | 5' TCCATTCATTCCCTCCTTGA 3' |
|  | Reverse | 5' TTTGCTGGACACCCTGGTA 3' |
| Rv3874 | Forward | 5' CCAAGAAGCAGCCAATAAGC 3' |
|  | Reverse | 5' CTGCTGCTGCTCCTCGTC 3' |
| Rv0692 | Forward | 5' GGCGACTACACCCACAGG 3' |
|  | Reverse | 5' AGGATGGTGCGATTTTTCAG 3' |
| Rv1703c | Forward | 5' TGTTCCTCGACCATGACAAG 3' |
|  | Reverse | 5' ATTGTCGGCAACCACGAT 3' |
| Rv2949c | Forward | 5' GCTAATGGCACCCTCACT 3' |
|  | Reverse | 5' TCGGTGAAACATCGTGAA 3' |
| Rv3134c | Forward | 5' GGAGGTGGACAATGGTGTG 3' |
|  | Reverse | 5' GTGTTTCAGCAGCGTGGAC 3' |
| Rv1735c | Forward | 5' GAGCGTCGGTGGTGTTCT 3' |
|  | Reverse | 5' GGACACGGTGGACGACAT 3' |
| 16S rRNA | Forward | 5'GTGGCGAACGGGTGAGTAAC 3' |
|  | Reverse | 5' ATGCATCCCGTGGTCCTATC 3' |
